# CRISPR/Cas9-induced knockout of an amino acid permease gene (*AAP6*) reduced *Arabidopsis thaliana* susceptibility to *Meloidogyne incognita*

**DOI:** 10.1186/s12870-024-05175-5

**Published:** 2024-06-08

**Authors:** Tushar K. Dutta, Katakam Rupinikrishna, Voodikala S. Akhil, Neeraj Vashisth, Victor Phani, Pankaj, Anil Sirohi, Viswanathan Chinnusamy

**Affiliations:** 1 Division of Nematology, ICAR-Indian Agricultural Research Institute, New Delhi, 110012 India; 2 Department of Agricultural Entomology, College of Agriculture, Uttar Banga Krishi Viswavidyalaya (UBKV), Balurghat, 733133 India; 3 Division of Plant Physiology, ICAR-Indian Agricultural Research Institute, New Delhi, 110012 India

**Keywords:** Mutation, *S* gene, *R* gene, Amino acid transporter, Multiplication ratio, Gene expression

## Abstract

**Background:**

Plant-parasitic root-knot nematode (*Meloidogyne incognita*) causes global yield loss in agri- and horticultural crops. Nematode management options rely on chemical method. However, only a handful of nematicides are commercially available. Resistance breeding efforts are not sustainable because *R* gene sources are limited and nematodes have developed resistance-breaking populations against the commercially available *Mi*-*1.2* gene-expressing tomatoes. RNAi crops that manage nematode infection are yet to be commercialized because of the regulatory hurdles associated with transgenic crops. The deployment of the CRISPR/Cas9 system to improve nematode tolerance (by knocking out the susceptibility factors) in plants has emerged as a feasible alternative lately.

**Results:**

In the present study, a *M. incognita*-responsive susceptibility (*S*) gene, amino acid permease (*AAP6*), was characterized from the model plant *Arabidodpsis thaliana* by generating the *AtAAP6* overexpression line, followed by performing the GUS reporter assay by fusing the promoter of *AtAAP6* with the *β-glucuronidase* (*GUS*) gene. Upon challenge inoculation with *M. incognita*, overexpression lines supported greater nematode multiplication, and *AtAAP6* expression was inducible to the early stage of nematode infection. Next, using CRISPR/Cas9, *AtAAP6* was selectively knocked out without incurring any growth penalty in the host plant. The ‘Cas9-free’ homozygous T_3_ line was challenge inoculated with *M. incognita*, and CRISPR-edited *A. thaliana* plants exhibited considerably reduced susceptibility to nematode infection compared to the non-edited plants. Additionally, host defense response genes were unaltered between edited and non-edited plants, implicating the direct role of *AtAAP6* towards nematode susceptibility.

**Conclusion:**

The present findings enrich the existing literature on CRISPR/Cas9 research in plant-nematode interactions, which is quite limited currently while compared with the other plant-pathogen interaction systems.

**Supplementary Information:**

The online version contains supplementary material available at 10.1186/s12870-024-05175-5.

## Background

As an important biotic stressor, plant-parasitic nematodes (PPNs) usurp global crop productivity to the tune of 200 billion US dollars (inflation-adjusted) economic loss per year [1, 2]. Polyphagous root-knot nematodes (RKN: *Meloidogyne* spp.) can infect more than 3000 genera of host plants [3]. The southern RKN *M. incognita* is considered a serious biotic threat to solanaceous and cucurbitaceous vegetable crops in tropical and subtropical countries, including India [4, 5]. During the parasitism process, RKNs secrete effector proteins from their pharyngeal glands that directly interact with plant proteins to initiate the induction of specialized feeding cells (referred to as giant cells) in the root vascular cylinder [6]. The metabolically active giant cells supply nutrients to the feeding RKNs for prolonged durations to facilitate the life cycle completion of these sedentary endoparasites [7]. The root tissues surrounding the giant cells become hypertrophied to form the macroscopic galls. RKN-induced galls seriously hamper normal plant physiology and ultimately affect crop yield [8].

PPN management options are extremely reliant on chemical methods, however, only a handful of nematicides such as fluensulfone and fluopyram are commercially available, with label claims for a limited number of target crops and nematodes [9]. A number of sustainable management strategies have been tested, including the generation of PPN-resistant plants via molecular breeding of resistance (*R*) genes [10] and via the adoption of the RNAi strategy [11]. However, transfer of *R* genes to the cultivated crop species from their wild relatives is a time-consuming process [12], and *M. incognita* has developed resistance-breaking phenotypes against the commercially available *Mi* gene (*R* gene)-expressing tomatoes [5]. Additionally, the most RNAi crops are yet to be commercialized because of the regulatory hurdles associated with transgenic crops [13, 14]. Deployment of the CRISPR/Cas9 system for improving plant tolerance against PPNs appears to be a feasible alternative because this strategy is less time-consuming and because it is non-transgenic (especially SDN (*s*ite-*d*irected *n*uclease)-1 and SDN-2 editing categories) can bypass the stricter regulatory guidelines [15].

The *R* gene-mediated resistance is reliant on the recognition of the corresponding nematode avirulence gene or effector [16, 17]. The dominantly-inherited *R* genes provide a narrow-spectrum of resistance that PPNs can occasionally overcome [18]. A number of PPN-specific susceptibility (*S*) genes have been identified from different host plants that facilitate PPN disease progression by either aiding in PPN penetration of host tissue (class 1), and/or negatively regulating the host immune system (class 2), and/or providing sustained metabolite supply to PPNs for their life cycle completion (class 3) [18, 19]. Since *S* genes are recessively inherited, knocking out the *S* genes via the CRISPR/Cas9 system can provide prolonged resistance in plants against PPNs, which cannot readily overcome the *S* gene-mediated resistance [18].

Although CRISPR/Cas9 knockout of *S* genes for improving disease (virus, bacteria, and fungi) tolerance in host plants has made considerable advancements [20], its application for achieving PPN tolerance is yet underexploited territory. The class 2 type *S* genes, *GmLMM1*, *SlWRKY45*, and *OsHPP04*, when knocked out via CRISPR/Cas9, improved resistance in soybean (cvs. Williams 82 and DN50), tomato (cv. Castlemart), and rice (cv. Nipponbare) against *M. incognita*, *M. incognita*, and *M. graminicola* was obtained, respectively [21–23]. CRISPR/Cas9 knockout of class 3 type *S* genes, *CsMS* and *SlARF8*, conferred reduced susceptibility in cucumber (cv. Xintaimici) and tomato (cv. Micro-Tom), respectively, against *M. incognita* infection [24, 25]. In the model plant *Arabidopsis thaliana*, when a *M. incognita*-responsive *S* gene, *AtHIPP27*, was knocked out via CRISPR/Cas9, improved RKN resistance was documented [18]. Based on the findings of these limited number of studies, it is imperative that more number of CRISPR/Cas9 research must be performed targeting various *S* genes to achieve a consensus understanding for future applications in plant nematology.

Since PPNs cannot synthesize the essential amino acids, their dietary requirement of amino acids is met by the feeding cells, which are enriched with different types of amino acid transporter (AAT) families [26]. One of the extensively studied AAT families is the amino acid permease (AAP) family [27, 28]. In *A. thaliana*, eight AAP paralogs (AAP1–8) were found to be involved in various steps of the amino acid transport mechanism [29, 30]. *AtAAP* genes were transcriptionally upregulated in *A. thaliana* upon infection with *M. incognita* [31] and cyst nematode (CN), *Heterodera schachtii* [32]. Among the AAP family, *AtAAP6* was greatly expressed in the giant cells [31] and syncytia (feeding cells induced by the CN; [33]). Using T-DNA insertional mutagenesis, the putative *S* gene function of *AtAAP* genes (mostly *AtAAP6*) was established in *A. thaliana*-*M. incognita*/*H. schachtii* pathosystems [26, 32, 34]. In root tissues, *AtAAP6* expression was localized to the vascular tissues, and localization patterns indicated its involvement in long-distance transport of amino acids [35]. Upregulated expression of *AAP6* was demonstrated in *H. glycines*-infected soybean roots [36]. A genome-wide association analysis (GWAS) showed the likely involvement of *TaAAP6* in wheat susceptibility to *H. filipjevi* [37].

As a proof-of-concept, the present study generated *AAP6* overexpression and promoter::*β-glucuronidase* (*GUS*) fusion *A. thaliana* lines to validate *AtAAP6*’s nematode-responsive nature. Next, *AAP6* knockout *A. thaliana* lines were generated via CRISPR/Cas9 to establish *AtAAP6*’s role in conferring reduced susceptibility to *M. incognita*.

## Results

### AAP6 orthologues are omnipresent in dicot plant families and *AAP6* is constitutively expressed in *A. thaliana*

**Fig. 1:**
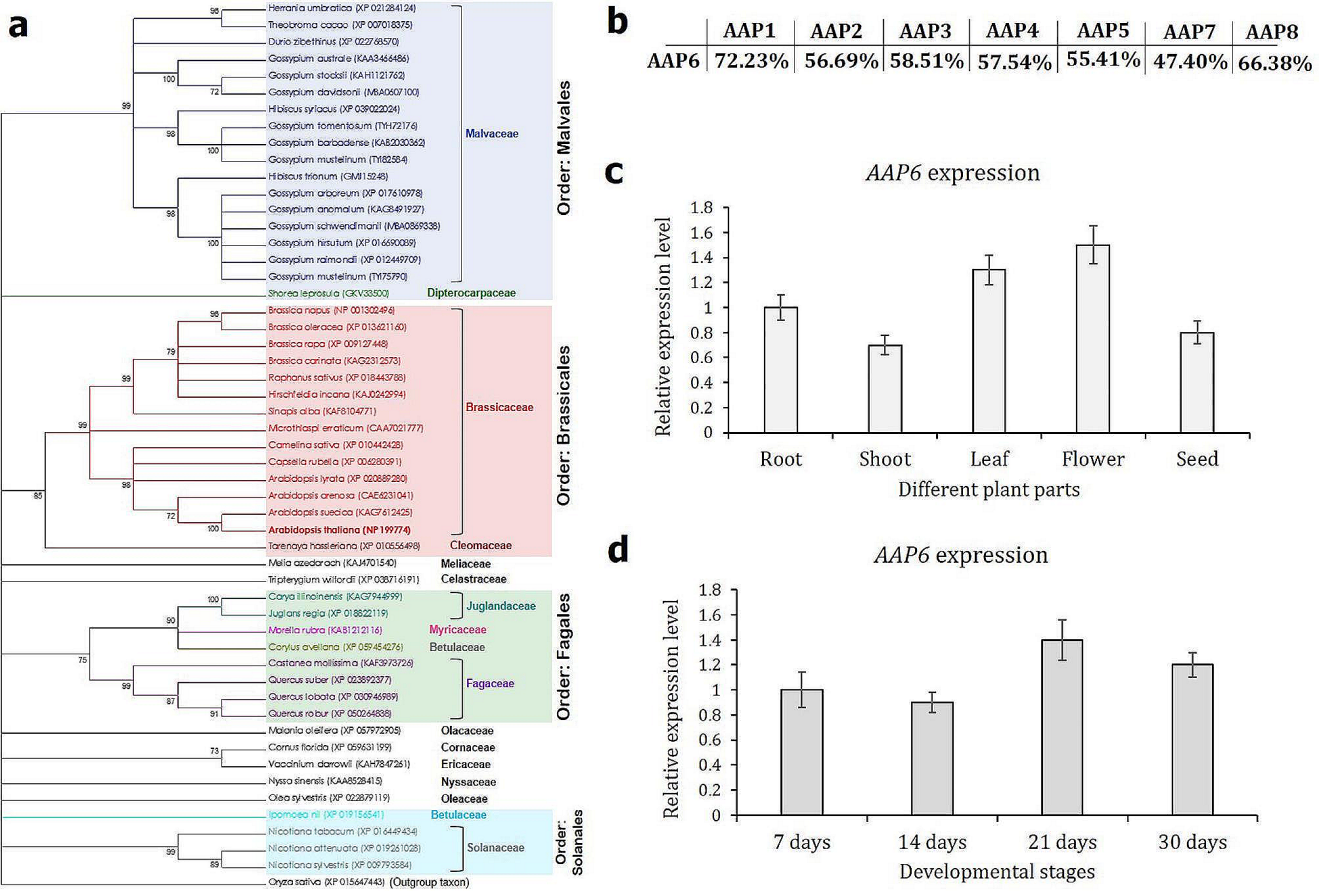
*AAP6* is greatly conserved in dicotyledonous plants and ubiquitously expressed in *A. thaliana*. **(a)** Evolutionary relationships of the AtAAP6 protein (entry is indicated in bold font) with its corresponding orthologues from other plant families. The phylogenetic tree was constructed using the ML method. Bootstrap consensus was inferred from 1000 replicates, and branches are supported by > 70% of replicates. The NCBI accession numbers of 52 different entries are provided in parentheses. The tree was rooted with the *O. sativa* subsp. *japonica* AAP6 protein as the out-group. Entries in different colors represent different plant families, as written aside the specific clusters. Solid rectangles in different colors indicate different plant orders. **(b)** The comparative amino acid sequence identity of AAP6 with its paralogues (AAP1, AAP2, AAP3, AAP4, AAP5, AAP7, and AAP8) from *A. thaliana*. **(c, d)** RT-qPCR-based expression analysis of the *AAP6* gene in different plant parts and developmental stages of *A. thaliana* ecotype Columbia-0. Fold change in expression was set at 1 in root tissue and 7 days-old-plant, and statistically compared with *AAP6* expression in other plant parts and developmental stages, respectively (no significant difference was observed; Tukey’s HSD test, *P* > 0.01). Gene expression was normalized using two housekeeping genes of *A. thaliana* (ubiquitin and *18 S rRNA*). Each bar represents the mean fold change value ± standard error (SE) of qPCR runs in five biological and three technical replicates

The amino acid sequence encoded by *AtAAP6* (Gene ID: AT5G49630, *A. thaliana* TAIR genome assembly) was used as a query in the BLASTp algorithm in the NCBI non-redundant database to identify the potential AAP6 orthologous sequences across the kingdom Plantae. AtAAP6 orthologous entries were obtained from 52 species encompassing 17 families of dicotyledonous plants with a high degree of sequence similarity (percent identity: 77.27–99.79%, query coverage: 92–100%, expect value: 0.0). To infer the evolutionary relationship among these sequences, a Maximum Likelihood (ML) method-based phylogenetic tree was constructed. The tree was rooted using the AAP6 sequence from *Oryza sativa japonica* as the outgroup. AtAAP6 formed a discreet clade with AAP6 sequences of 14 Brassicaceae family members (Fig. 1a). AAP sequences corresponding to other families, including Malvaceae, Solanaceae, Fagaceae, and Juglandaceae branched away from the Brassicaceae clade (Fig. 1a). Intriguingly, families belonging to identical orders (Brassicales, Malvales, Fagales, and Solanales) branched nearer (Fig. 1a), indicating the plant order-specific sequence conservation of the AAP6 protein. Within the *A. thaliana* genome, AAP has eight paralogs (AAP1–8). A pairwise sequence comparison indicated that AAP6 has 47.40–72.23% amino acid sequence identity with its paralogous sequences (Fig. 1b). Pairwise sequence alignments showed a discontinuous stretch of sequence identity between AAP6 and its closest homologue AAP1 (supplementary Fig. 1), indicating that AAP6 is unique in its identity across the AAP paralogs.

The expression profile of *AAP6* in various developmental stages and plant parts of *A. thaliana* was analyzed using RT-qPCR. *AtAAP6* expression did not significantly alter (*P* > 0.01) across the plant parts (root, shoot, leaf, flower, and seed; Fig. 1c) and whole plant developmental stages (7, 14, 21, and 30 days; Fig. 1d), suggesting the ubiquitous expression of *AAP6* in *A. thaliana*.

### *AtAAP6* expression is inducible to *M. incognita* infection in *A. thaliana*

**Fig. 2:**
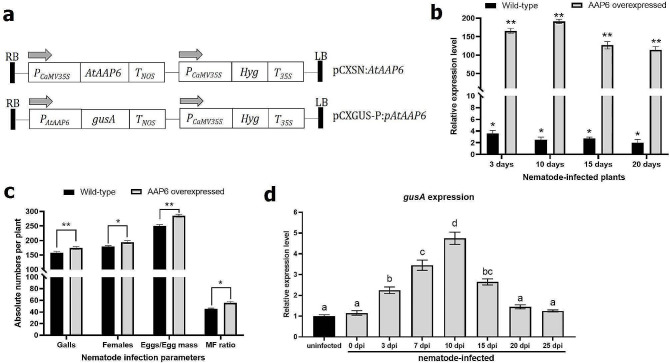
*AtAAP6* overexpression increased *A. thaliana* susceptibility to *M. incognita*. **(a)** Schematic representation of the T-DNA regions corresponding to the *AtAAP6* overexpression vector (driven by the CaMV35S promoter) and promoter::*GUS* fusion vector (*gusA* expression is driven by the promoter of the *AtAAP6* gene). *T*_NOS_, *T*_*35S*_ – polyadenylation signals of nopaline synthase and CaMV35S for transcription termination. Arrows indicate the direction of transcription. Hygromycin (*Hyg*) was used as the selectable marker. LB, RB – left and right borders. **(b)** RT-qPCR-based detection of an increase in *AtAAP6* mRNA abundance in an overexpression line at 3, 10, 15 and 20 days post inoculation (dpi) of *M. incognita*. Asterisks (*P < 0.01, **P < 0.0001; paired *t*-test) represent significant differential expression of the *AtAAP6* transcript in nematode-infected wild-type and overexpression line when compared to the baseline expression in uninfected control plants (fold change values were set at 1). Gene expression data was normalized using two reference genes, i.e., *A. thaliana 18 S rRNA* and ubiquitin. Bars represent the mean fold change value of five biological and three technical replicates ± standard errors. **(c)** Numbers of gall, female, egg per egg mass, and MF ratio per root system were significantly higher in the overexpression line than the wild-type at 30 dpi. Bars represent the mean of five replications ± standard errors. Asterisks indicate a significant difference between two treatments (*P < 0.05, **P < 0.01; two-way ANOVA followed by Tukey’s significant difference test). **(d)** RT-qPCR-based validation of *gusA* gene expression in a transformed line (harboring the promoter::*GUS* fusion) at 0, 3, 7, 10, 15, 20 and 25 dpi. The fold change in gene expression was set at 1 in the uninfected control and compared with other treatments. Other parameters were kept as identical as described above. Bars with different letters are significantly different at *P* < 0.01, one-way ANOVA followed by Tukey’s test

Initially, using a zero background TA-cloning vector, *AtAAP6* (driven by the CaMV35S promoter; Fig. 2a) overexpression lines were generated in the *A. thaliana* Columbia-0 (Col-0) background. At 3, 10, 15, and 20 days after *M. incognita* inoculation, 165-, 191-, 126-, and 113-fold significant upregulation (*P* < 0.0001) in the *AtAAP6* transcript level was observed in T_3_ plants of a homozygous overexpression line, respectively, compared to the uninfected plants (Fig. 2b). Further, an increased nematode infection level was documented in the overexpression line, indicating the *M. incognita*-responsive nature of the *AtAAP6* gene. At 30 dpi, the number of galls, females, eggs per egg mass, and MF ratio were significantly increased by 10.44% (*P* < 0.01), 8.11% (*P* < 0.05), 14.17% (*P* < 0.01), and 23.41% (*P* < 0.05) in the overexpression line compared to the wild-type plant, respectively (Fig. 2c).

**Fig. 3:**
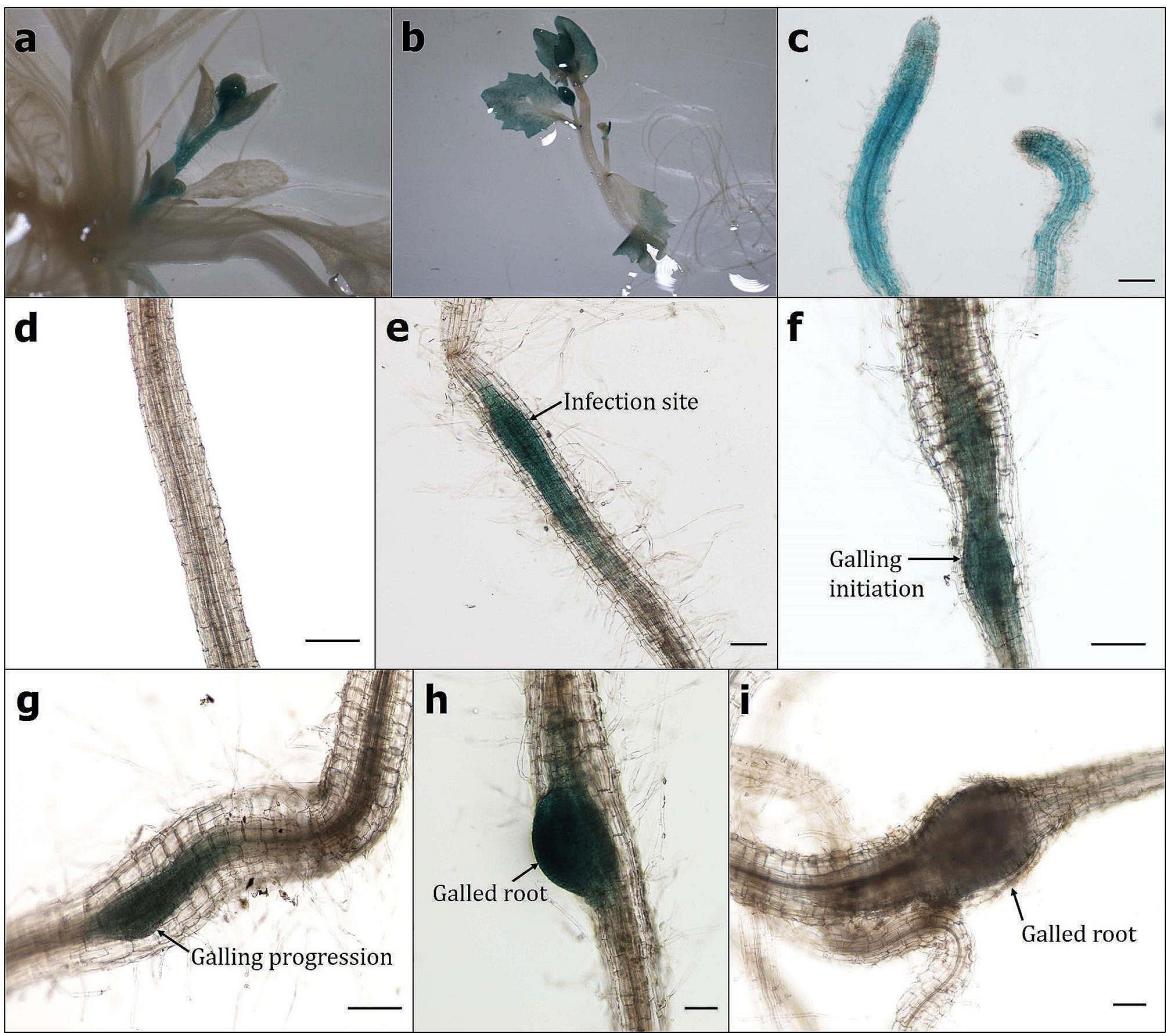
Strong and localized expression of *AtAAP6* in the galled root of *A. thaliana* upon *M. incognita* infection. Expression of *pAAP6::GUS* in the growing tissues of a three-week-old plant: (**a**) shoot, (**b**) leaf, (**c**) root. Expression of *pAAP6:GUS* in nematode-infected root segments at different days post inoculation (dpi): (**d**) 0 dpi, (**e**) 3 dpi, (**f**) 7 dpi, (**g**) 10 dpi, (**h**) 15 dpi, (**i**) 20 dpi Scale bar = 100 μm

To validate the hypothesis that *AtAAP6* expression is *M. incognita* infection-inducible, the *A. thaliana* Col-0 plant was transformed with the *P*_*AtAAP6*_::*GUS* construct, in which the promoter region of the *AtAAP6* gene was fused with the *GUS* reporter gene (Fig. 2a). T_3_ plants of a homozygous line were challenge inoculated with *M. incognita*, and *gusA* gene expression was assessed in nematode-infected roots at different time points. Compared to the uninfected root, *gusA* expression was significantly (*P* < 0.01) elevated at 3 dpi, reached its peak at 10 dpi, and showed elevated (*P* < 0.01) expression till 15 dpi in the nematode-infected roots (Fig. 2d). However, at 20 and 25 dpi, *gusA* expression did not significantly differ between uninfected and nematode-infected roots (Fig. 2d), indicating that *AtAAP6* expression is putatively responsive to the early infection stage of *M. incognita*. To validate this, histochemical GUS activity was analyzed in the nematode-infected root segments. GUS staining was visible in the growing leaf, shoot and root tissues of uninfected plants (Fig. 3a-c), suggesting the probable localization of *AtAAP6* to amino acid sink tissues. Compared to the no staining in root vasculature at 0 dpi (Fig. 3d), intense GUS staining was observable at the nematode infection site in the vascular tissue (the location of giant cell induction) at 3 dpi (Fig. 3e). GUS staining became highly localized in the infection site and adjacent root vascular tissue during galling initiation at 7 dpi and in moderately galled roots at 10 and 15 dpi (Fig. 3f-h). As expected, GUS staining was not detectable in the galled root at 20 dpi (Fig. 3i).

### Generation of genome-edited *A. thaliana* lines via targeted knockout of *AtAAP6* gene

**Fig. 4:**
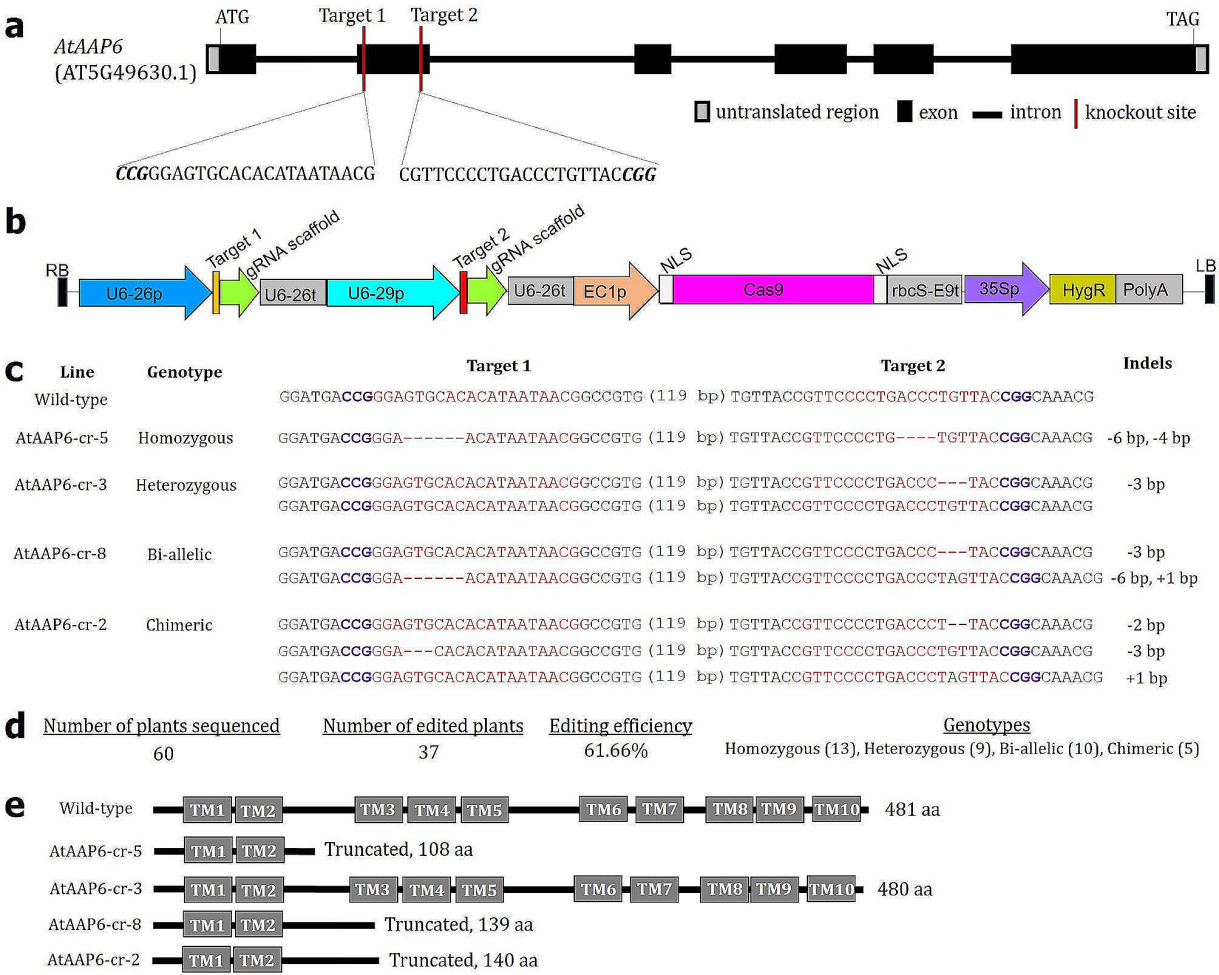
Targeted knockout of the *AtAAP6* gene in *A. thaliana* using the CRISPR/Cas9 system. **(a)** Two gRNA spacer sequences (target 1 and 2 are in the negative and positive strand, respectively) were designed from exon 2 of the *AtAAP6* gene. Protospacer adjacent motif (PAM) sites are bold and italicized. **(b)** The T-DNA portion of the recombinant Cas9-expressing vector (pHEE401:*AtAAP6*) is schematically illustrated. Two gRNA expression cassettes (guide RNA + scaffold) were assembled via Golden Gate cloning. gRNA and Cas9 expression were driven by the *Arabidopsis* U6 promoter and an egg cell-specific promoter (EC1p), respectively. NLS – nuclear localization signal, 35Sp – CaMV35S promoter, HygR – Hygromycin resistance, LB, RB – left and right borders. **(c)** Sequencing-based identification of edited events in the T_0_ generation of plants. Four different types of mutations, i.e., homozygous, heterozygous, bi-allelic, and chimeric were detected with a maximum deletion (red hyphens) and insertion (green letter) of 6 and 1 bp, respectively. **(d)** Summarized data shows the editing efficiency and specific number of different mutant genotypes obtained. **(e)** The predicted mutated proteins in different events are schematically represented. AtAAP6 was heavily truncated (due to premature translation termination) in a homozygous event, AtAAP6-cr-5. TM – transmembrane domains

The *AtAAP6* genomic sequence (corresponding to only one transcript variant, AT5G49630.1) contains six exons and five introns (Fig. 4a). Since targeted knockout towards the 5´ end of a gene ensures a greater probability of obtaining the truncated peptide, gRNA designing was initially attempted from *AtAAP6* exon 1. However, appropriate gRNA spacer sequences could not be designed from exon 1, the sequence of which was quite short, and gRNA secondary structures were not ideal. Two gRNA spacers were designed from exon 2 (Fig. 4a) with no off-target sites (with at least 3 or 4 nucleotide mismatches) across the *A. thaliana* genome. The secondary structure of both gRNAs harbored maximum free guide sequence (minimal internal base pairing in the guide sequence of crRNA results in greater target recognition), a stable tetra loop (connects crRNA to tracrRNA), and stem loops 2 and 3 (in tracrRNA). Stem loops promote Cas9-gRNA-target DNA complex formation that ultimately aids in improving the in vivo editing efficiency (supplementary Fig. 2). The editor plasmid pHEE401:*AtAAP6* expressing the gRNA cassettes (two gRNAs and their scaffolds driven by *Arabidopsis* U6 promoter), *Cas9* (driven by *Arabidopsis* egg cell-specific promoter), and antibiotic resistance gene *Hyg* (driven by CaMV35S promoter) was mobilized into *R. radiobacter* strain GV3101, which was transformed into *A. thaliana* (Fig. 4b).

Ten independent T_0_ lines (each containing six clonal lines) were generated (named AtAAP6-cr-1 to AtAAP6-cr-10) and genotyped via Sanger sequencing. Four different types of insertion-deletion (indel) mutations (homozygous, heterozygous, bi-allelic, and chimeric) were detected in the target sites of different T_0_ lines (Fig. 4c; supplementary Fig. 3). Taken together, an editing efficiency of 61.66% was obtained for the *AtAAP6* gene (Fig. 4d). In the wild-type, the encoded AtAAP6 protein (481 amino acids long) contained 10 transmembrane (TM) domains (Fig. 4e). Although in the mutant line AtAAP6-cr-3, encoded AtAAP6 (480 aa) was shorter, the reading frame of AtAAP6 was not disrupted. In the mutant lines AtAAP6-cr-2, AtAAP6-cr-5, and AtAAP6-cr-8, truncated peptides of 140, 108, and 139 aa were predicted, respectively, due to the premature translation termination of AtAAP6 (Fig. 4e). The homozygous mutant line AtAAP6-cr-5 was taken forward for further studies.

Next, the expression level of the *AtAAP6* transcript was assessed in line AtAAP6-cr-5 via RT-qPCR. Notably, *AtAAP6* expression was significantly downregulated (*P* < 0.01) in the edited line compared to the wild-type plants (supplementary Fig. 4). Expression of paralogous genes such as *AtAAP1*, *AtAAP2*, *AtAAP3*, *AtAAP4*, *AtAAP5*, *AtAAP7*, and *AtAAP8* was not significantly altered (*P* > 0.01) between the wild-type and edited line (supplementary Fig. 4), confirming the targeted knockout of the *AtAAP6* gene.

To obtain the ‘Cas9-free’ homozygous mutant line, T_2_ plants (the *Cas9* gene might have been segregated out in a few of the progeny plants) were generated from T_1_, which was generated from T_0_ by self-pollination. The presence or absence of a *Cas9* gene-specific fragment (amplified using *Cas9*-specific primer) and a reference gene *18 S rRNA*-specific fragment (amplified using *18 S*-specific primer) were detected in 13 progeny plants of the AtAAP6-cr-5 T_2_ line. Plant numbers 4, 7, and 10 were considered the ‘Cas9-free’ plants since these plants did not amplify the *Cas9* fragment but amplified the reference gene fragment (supplementary Fig. 5).

### Loss of function of AtAAP6 reduced *A. thaliana* susceptibility to *M. incognita* without altering the plant basal defense

**Fig. 5:**
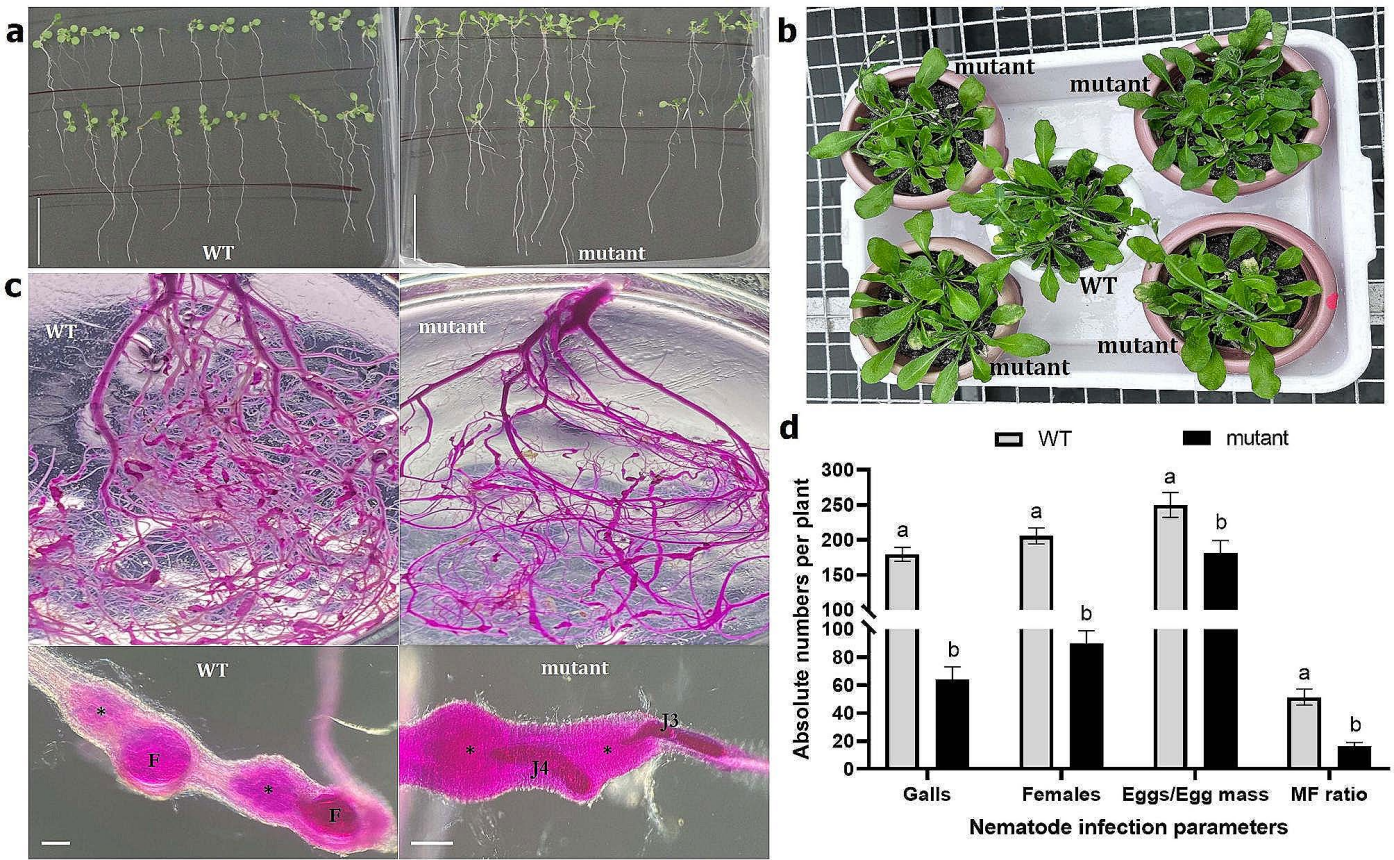
Targeted mutagenesis of *AtAAP6* conferred improved resistance in *A. thaliana* against *M. incognita* infection. **(a)** Growth phenotypes of wild-type (WT) and mutant plants at two weeks after germination in MS agar. Scale bar = 3 cm. **(b)** Shoot morphology of WT and mutant plants at 30 days after germination in soil. **(c)** Photomicrographs depict lower galling intensity in a mutant root system compared to the WT root system at 30 days post inoculation (dpi) of nematodes. Magnified images at the bottom show the developmental delay of nematodes in mutant roots because WT roots harbored females (F), whereas mutants harbored J3/J4 spike-tail stages at that time. Asterisks indicate the location of putative feeding cells. Scale bar = 500 μm. **(d)** Numbers of gall, female, egg per egg mass, and MF ratio per root system were reduced in mutants compared to WT at 30 dpi. Bars represent the mean of five replications ± standard error. Different letters indicate a significant difference (within a specific infection parameter) at *P* < 0.0001, a two-way ANOVA followed by Tukey’s significant difference test

The T_3_ generation of ‘Cas9-free’ homozygous AtAAP6-cr-5 plants was assessed for their growth phenotypes, followed by the challenge inoculation with *M. incognita* J2s in the pots. The average dry weight and average root length of a 14-day-old seedling, the average height of a 30-day-old plant, and the average flowering time did not significantly differ (*P* > 0.01) between the wild-type and edited line (Fig. 5a, b; supplementary Fig. 6), indicating that the induced mutation of *AtAAP6* did not cause any growth penalty or pleiotropic effects in *A. thaliana*. Upon nematode infection at 30 dpi, considerably lower galling intensity was documented in the mutant root system compared to the wild-type root system. Additionally, a developmental delay in *M. incognita* life cycle progression was observed in mutant roots because, while wild-type roots supported mature females at 30 dpi, mutant roots supported the third/fourth stage juveniles (J3/J4) or spike-tail stages (Fig. 5c). The improved *M. incognita* resistance in the edited line was additionally validated by analyzing the different nematode infection parameters. The average numbers of gall, female (representing an equal egg mass), egg per egg mass, and MF ratio were significantly reduced (*P* < 0.0001) by 64.29, 56.28, 27.44, and 68.23% in the edited line, respectively, compared to the wild-type plants (Fig. 5d).

To assess whether the improved nematode resistance of the edited line is related to the enhanced host basal defense responses, the RT-qPCR-based transcriptional profile of defense marker genes was analyzed in the root and shoot tissues of *M. incognita*-infected wild-type and AtAAP6-cr-5 plants at 3 dpi. Ten marker genes were targeted that belonged to different categories, such as oxidative stress (peroxidase, *AtMPK4*), the salicylic acid pathway (*AtEDS1*, *AtPAD4*, *AtPR1*, *AtPR2*), the jasmonic acid pathway (*AtPDF1.2*, *AtHEL1*), and ethylene signaling (*AtERF6*, *AtACS2*). The relative expression of neither of the targeted defense genes significantly altered (*P* > 0.01) between the wild-type and edited line (supplementary Fig. 7), implicating the key role of *AAP6* in modulating *A. thaliana* susceptibility to *M. incognita*.

## Discussion

In the current study, using CRISPR/Cas9 knockout, we demonstrated that *AAP6* is an important susceptibility factor for *M. incognita* infection in *A. thaliana*. Earlier, using T-DNA mutations, *AAP1*, *AAP2*, and *AAP6* were shown to be involved in *H. schachtii* parasitism in *A. thaliana* [32]. Similarly, T-DNA mutations in *AAP3* and *AAP6* indicated their association with *M. incognita* parasitic success in *A. thaliana* [26]. Being considered one of the most important AAT families, AAPs are extensively involved in plant-pathogen interactions [30]. CRISPR/Cas9 knockout of *SlAAP5* in tomato conferred improved resistance against the hemi-biotroph oomycete pathogen *Phytophthora infestans* [27]. TILLING-induced mutations in *CsAAP2* caused reduced susceptibility of cucumber plants to the obligate biotrophic oomycete pathogen *Pseudoperonospora cubensis* [27]. Root cell layer-specific abundance of *AAP* transcripts was observed in *A. thaliana* upon infection of hemi-biotrophs *Phytophthora parasitica* and *Verticillium longisporum* [38]. For example, *AtAAP3*, *AtAAP5*, and *AtAAP6* were induced in the stele, and *AtAAP6* was additionally expressed in the cortex, upon *P. parasitica* infection. *AtAAP4* was upregulated in the cortex upon *V. longisporum* infection [38].

Our phylogeny analysis showed that AAP6 orthologues are quite omnipresent across the dicotyledonous plant families, and a plant order-specific sequence conservation of the AAP6 protein was also documented. This suggests that *AAP6* can be exploited as an important *S* gene target in cultivated crop species (such as cotton, *Gossypium* spp.) to obtain *M. incognita* resistance by deploying the CRISPR/Cas9 strategy. Interestingly, *AAP* genes have been placed into either class 2 or class 3 types of *S* genes. *AAP*s can act as negative regulators of plant defense responses against hemi-biotropic pathogens [30]. For obligate biotrophs, *AAP*s provide a sustained supply of accessible amino acids to the feeding pathogen via creating an artificial sink [27]. In plant-PPN interactions, most *AAP*s are putatively of class 3 type because they facilitate the sustained metabolite supply to the feeding RKNs and CNs by establishing the giant cells and syncytia, respectively [18, 26, 32]. In our qPCR analysis, *AtAAP6* was ubiquitously expressed in different plant parts and developmental stages of *A. thaliana*. Notably, AAPs are the one-directional transporters that transport amino acids to different growing plant parts across the xylem and phloem vessels [30, 35].

To validate the *M. incognita*-responsive nature of the *AtAAP6* gene, we initially generated the *AAP6* overexpression line in *A. thaliana* and challenge-inoculated the plants with *M. incognita*. Nematode infection levels (in terms of gall numbers, endoparasitic females, and nematode fecundity) were significantly enhanced in the overexpression line compared to the wild-type plants, exemplifying the putative correlation between *AtAAP6* overexpression and *M. incognita* susceptibility. Next, a GUS reporter assay was conducted in which *A. thaliana* was transformed with the promoter of *AtAAP6* fused to the *GUS* reporter gene. *GUS* gene expression was considerably increased in the nematode-infected roots during the early stage of the *A. thaliana*-*M. incognita* interaction, i.e., 3, 7, 10, and 15 dpi. In corroboration, intense and localized GUS staining was observed in the infection site and galled tissue of nematode-infected roots during 3, 7, 10 and 15 dpi. We assumed that *AtAAP6* gene expression is inducible to the early infection stage of *M. incognita*. In an earlier study, using promoter::GUS fusion, *AtAAP6* expression was localized to the entire gall, including giant cells, at 14 days after *M. incognita* infection [31]. Conversely, in another GUS reporter assay, *AtAAP6* expression remained quite strong even during the late infection stage of *M. incognita*, i.e., four weeks after inoculation [26]. The differences in expression localization patterns suggest that *AtAAP6* may act in variable manners to transport amino acids to the nematode feeding sites. In our study, GUS staining was also detected in the growing shoot and root of the uninfected plant. This aligns with the previous findings where GUS activity (corresponding to *AtAAP6*) was localized to the leaf vascular tissue and lateral roots [26, 31, 39].

Using CRISPR/Cas9, we selectively knocked out the *AAP6* gene (the encoded AAP6 protein was truncated due to premature translation termination) in *A. thaliana*, and a ‘Cas9-free’, homozygous T_3_ line was generated without incurring any growth penalty or pleiotropic effects (due to an induced mutation in the *AtAAP6* gene) in the host plant. Using qPCR, targeted knockout of *AtAAP6* was also confirmed because expression of other *AAP* paralogs was unaffected in the mutant line. Upon challenge inoculation, genome-edited plants exhibited significantly reduced susceptibility to *M. incognita*, compared to the wild-type plants. At 30 dpi, a significantly reduced number of galls, females, eggs per egg mass, and MF ratio (which determines the nematode reproductive success) were recorded in the edited line compared to the wild-type roots. Additionally, a developmental delay in the nematode life cycle progression was observed in the mutant root compared to the wild-type ones. The basal defense response of the edited line and wild-type plant (root and shoot tissues were separately analyzed) upon *M. incognita* infection was analyzed using qPCR at 3 dpi. The relative expression level of the ten defense marker genes was unaltered between the edited line and wild-type plants, indicating the reduced nematode susceptibility of the genome-edited line is correlated to the targeted knockout of the *AtAAP6* gene rather than any indirect effect of altered host defense responses. Consistent with our finding, host defense genes were not differentially expressed between wild-type and CRISPR/Cas9-edited tomato plants (a class 3 type *S* gene *SlARF8* was knocked out) upon *M. incognita* infection [25]. On the contrary, host defense responses such as induction of defense genes (*OsKS4*, *OsPAL4*, *OsEDS*, *OsPR1a*, and *OsPR4*), reactive oxygen species (ROS) burst, and callose deposition were enhanced upon *M. graminicola* infection in the *OsHPP04* knocked out (via CRISPR/Cas9) rice line, indicating that *OsHPP04* is a class 2 type of *S* gene [23]. *OsHPP04* is a heavy metal-associated plant protein harboring a typical heavy metal binding (HMA) domain that contains a conserved Cys-X-X-Cys motif; Cys residues have copper binding specificity [40]. Interestingly, another type of HMA domain containing protein, i.e., heavy metal-associated isoprenylated plant protein (HIPP27), contains an additional C-terminal isoprenylation motif [41]. *HIPP27* has been classified as the class 3 type of *S* gene [18], and loss of function of *AtHIPP27* either via T-DNA mutation [41] or CRISPR/Cas9 [2] in *A. thaliana* did not alter the plant basal defense responses.

Compared to the quite extensive literature on other plant-pathogen interactions [20, 42, 43], CRISPR/Cas9 knockout of *S* genes for improving PPN tolerance in host plants is yet an underexploited research area. The findings of the present study enrich the existing literature by investigating the *S* gene function in a model plant. Expanding the repertoire of putative PPN-responsive *S* gene candidates will aid in translating the CRISPR research into agriculturally-important crop plants for obtaining PPN tolerance. However, the possibility of pleiotropic effects (due to *S* gene knockout) cannot be ignored because disrupting the function of a plant endogenous gene may lead to growth impairment in host plants. For example, CRISPR/Cas9 knockout of *SlARF8* (*M. incognita*-responsive *S* gene) caused the phenotypic abnormality in *Solanum lycopersicum* roots [25]. CRISPR/Cas9 knockout of *OsPR10* (a defense gene responsive to *M. graminicola* infection) shortened the plant height in *Oryza sativa* [44]. As an alternative, the promoter region of the *S* gene may be targeted for selectively switching off the gene function since the same strategy has been successfully used to arrest *Xanthomonas oryzae* infection in rice by targeting the *SWEET* gene promoters [45, 46]. In addition, precise editing tactics such as prime editing [47] can be adopted to introduce point mutations in the susceptible allele of a *R* gene that confers PPN resistance. The majority of the putative *S* genes (characterized via GWAS, transcriptome analysis, overexpression, RNAi, and T-DNA mutation) identified from different plant-PPN pathosystems are of ‘class 3 types’ that may have redundant functions in the PPN parasitism processes [18]. CRISPR-induced mutagenesis of these *S* genes in agriculturally-important crop plants may render the crop vulnerable to untargeted PPNs. Similar phenomena were already reported in different plant-pathogen interactions [48, 49]. In the future, a greater number of PPN-responsive *S* genes must be characterized using the CRISPR/Cas9 system, and their functional redundancy should be investigated by deploying the multiplex editing system.

## Materials and methods

### Bioinformatics of *AAP* genes

Sequences of *AAP6* and its paralogs were obtained from the *A. thaliana* TAIR genome assembly (https://plants.ensembl.org/Arabidopsis_thaliana/). *AAP6* orthologous sequences were obtained from the NCBI non-redundant database. Different sequence features of *AtAAP6*, including cDNA, coding sequence (CDS), exon, intron, 5´ and 3´ untranslated region (UTR), encoded amino acids, were analyzed in FGENESH (https://www.softberry.com/) and Expasy (https://web.expasy.org/) webservers. Conserved domains and motif signatures of the AtAAP6 protein were examined in the InterProScan (https://www.ebi.ac.uk/interpro/) database. AAP6 orthologous sequences from different plant species were aligned using the ClustalW multiple sequence alignment tool. A phylogenetic tree was constructed in MEGAX software by using the ML method and the Tamura 3-parameter model. The tree was generated based on the greatest log likelihood, and a discrete Gamma distribution was followed to model evolutionary rate differences between sites.

### Culture of *M. incognita*

A pure culture of *M. incognita* (Kofoid & White) Chitwood race 1 (confirmed by female perineal patterns and using a species-specific SCAR-PCR molecular marker) was maintained in the roots of tomato (*Solanum lycopersicum* cv. Pusa Ruby) in pots in the greenhouse at 28 ºC, 60% relative humidity (RH) with 16 h light and 8 h dark photoperiod (light level: 250 µmol photons m^− 2^ s^− 1^). Plants were harvested two months after *M. incognita* inoculation, and roots were cleaned free of soil. *M. incognita* egg masses extracted from the roots (using sterilized forceps) were hatched in sterile water at room temperature for 24–48 h. Readily-hatched second-stage juveniles (J2s) were used for infection experiments.

### *A. thaliana* growth conditions and challenge inoculation with *M. incognita*

*A. thaliana* ecotype Columbia-0 (Col-0) seeds were surface-sterilized with 70% ethanol, 0.1% HgCl_2_ and 0.1% SDS for 2, 5 and 5 min, respectively, followed by rinsing in sterile distilled water five times (for 2 min each). Seeds were germinated in Petri dishes containing half-strength Murashige and Skoog (MS) agar (Sigma Aldrich). Dishes were incubated in growth chambers at 21ºC, 60% RH with 16 h light/ 8 h dark at 150 µmol photons m^− 2^ s^− 1^. Fortnight-old seedlings were transplanted to 6-inch diameter pots containing 500 g soil rite (Keltech Energies Ltd., Bengaluru). Plants were grown in the regulated environment (at the National Phytotron Facility, Indian Agricultural Research Institute) at 21ºC, 60% RH with 16 h light/ 8 h dark at 150 µmol photons m^− 2^ s^− 1^. After three weeks of transplantation, each plant was inoculated with 1000 J2s of *M. incognita* near the root zone using a sterilized pipette tip. At 30 days post inoculation (dpi), plants were harvested to analyze the different nematode infection parameters, including numbers of gall, female (equivalent to an egg mass), eggs per egg mass, and multiplication factor (MF) ratio [(number of egg mass × number of eggs per egg mass) ÷ primary inoculum level]. The same procedure was adopted to examine the *M. incognita* infection level in transgenic (overexpression) and mutant (CRISPR-edited) lines. Ten plants were included in each treatment, and the whole experiment was repeated three times.

### Generation of *AtAAP6*-overexpressing *A. thaliana* lines

Total RNA was isolated from the fortnight-old *A. thaliana* leaves using the NucleoSpin RNA Plant Kit (TaKaRa) by following the manufacturer’s protocol. RNA was reverse-transcribed to cDNA via the SuperScript VILO cDNA synthesis Kit (Invitrogen). The CDS of *AtAAP6* (1446 bp) was PCR-amplified (primer details given in supplementary Table 1) from the cDNA using proofread-efficient Phusion DNA polymerase (Invitrogen). A zero-background TA cloning vector pCXSN [50, 51] was used for overexpressing *AtAAP6* (driven by the CaMV35S promoter). For this, pCXSN was digested with *Xcm*I (New England Biolabs) to generate T-overhangs, and A-overhangs were generated in PCR products via the A-tailing procedure (https://www.promegaconnections.com/a-quick-method-for-a-tailing-pcr-products/). Gel-purified PCR products were ligated to digested pCXSN using T4 DNA ligase (Invitrogen) with a standard insert to vector molar ratio of 3:1. The recombinant pCXSN:*AtAAP6* plasmid was transformed into *Escherichia coli* DH5α cells via electroporation. Post sequence verification, pCXSN:*AtAAP6* was transformed into *Rhizobium radiobacter* strain GV3101 by the freeze-thaw method.

For promoter::*GUS* fusion, genomic DNA was isolated from the fortnight-old *A. thaliana* leaves using the NucleoSpin Plant II Kit (TaKaRa) by following the manufacturer’s protocol. The promoter region of *AtAAP6* (1 Kb upstream of the start codon) was PCR-amplified (primer details given in supplementary Table 1) from the genomic DNA and cloned into the *Xcm*I-digested pCXGUS-P vector [50, 51] via TA cloning as explained earlier. The pCX-GUS-P:*AtAAP6* plasmid was transformed into *R. radiobacter* strain GV3101, as explained earlier.

A month-old *A. thaliana* Col-0 wild-type plants growing in pots were separately transformed with *R. radiobacter* harboring pCXSN:*AtAAP6* and pCX-GUS-P:*AtAAP6* by the floral dip method [52]. Plants were harvested during pod stage, T_0_ seeds were collected, sterilized, and germinated on MS media supplemented with the antibiotic hygromycin at 25 mg L^− 1^. Transgenic plants (3–4 leaf stage) that survived on medium containing hygromycin were transplanted to the pots containing soil rite in a growth chamber. T_3_ homozygous plants (growing in 500 g soil rite) were infected with 1000 *M. incognita* J2s, and different nematode infection parameters were assessed as described above.

### Histochemical GUS assay

5-bromo-4-chloro-3-indolyl-b-D glucuronide (X-Gluc) was used as the substrate to analyze the GUS activity in *M. incognita*-infected roots at different time points, such as 0, 3, 7, 10, 15, and 20 dpi. Harvested roots were carefully washed free of soil and immersed in the readily-prepared GUS staining solution (0.5 mM X-Gluc, 0.1 M Na_2_HPO_4_, 0.5 mM K_3_Fe(CN)_6_, 0.5 mM K_4_Fe(CN)_6_, 0.01 M EDTA, 20% methanol, and 0.1% Triton X-100) for 18 h at 37ºC. The clearing of root tissues was performed by replacing the solution with 70% ethanol. GUS-stained roots were observed under a Zeiss Axiocam MRm microscope, and images were obtained using a Carl Zeiss camera.

### Expression analysis of candidate genes

Total RNA was isolated from *M. incognita*-infected and control roots, as explained earlier. RNA integrity was examined via electrophoresing on 1% (w/v) agarose gel. RNA purity and quantity were assessed in a Nanodrop spectrophotometer (Thermo Fisher Scientific). Using the SuperScript VILO cDNA synthesis Kit (Invitrogen), ~ 1 µg RNA was reverse-transcribed into cDNA. qPCR-based expression analysis of various candidate genes (primer details given in supplementary Table 1) was performed in a CFX96 thermal cycler (BioRad). The PCR efficiency of each primer pair was calculated by using different primer concentrations in different RT-qPCR reactions, followed by generating the standard curve [53]. 10 µL of RT-qPCR reaction volume constituted 1.5 ng cDNA, 750 nM each of sense and antisense primers, and 5 µL SYBR Green PCR master-mix (BioRad). The RT-qPCR amplification condition was maintained as – a hot start phase of 95 °C for 30 s, 40 cycles of 95 °C for 10 s, and 60 °C for 30 s. Further, a melt curve program (95 °C for 15 s, 60 °C for 15 s, followed by a slow ramp from 60 to 95 °C) was added to visualize the specificity of RT-qPCR amplification. Quantification cycle (Cq) values were obtained from CFX Maestro software (BioRad). *A. thaliana* housekeeping genes, *18 S rRNA* and ubiquitin, were used as internal references to normalize the target gene expression. Fold change in gene expression was quantified using the 2^−ΔΔCq^ method. RT-qPCR runs comprised five biological and three technical replicates for each sample.

### gRNA designing for CRISPR/Cas9 assay

Using the *AtAAP6* sequence as the query, potential guide RNA (gRNA) spacer sequences (20 bp) accompanied by the protospacer adjacent motif (PAM) sequence (5´-NGG-3´) were searched across the *A. thaliana* genome using various gRNA designing tools, including RGEN (https://www.rgenome.net/), CHOPCHOP (https://chopchop.cbu.uib.no/), CRISPick (https://portals.broadinstitute.org/), CRISPR-PLANT (http://omap.org/crispr/), and MMEJ-KO (http://skl.scau.edu.cn/mmejko/). gRNAs that were commonly predicted by these tools were shortlisted. Other criteria followed while designing gRNA include a greater out-of-frame score (which predicts frame shift in the CDS), a greater microhomology score (which predicts double-strand break repair via microhomology-mediated end joining), and minimum self-complementarity within the targeted sequence. To avert any possibility of an off-target effect, shortlisted gRNAs were screened through the *A. thaliana* genome to identify any potential off-target sites. Finally, secondary structure prediction of gRNAs was performed in the RNAfold webserver (http://rna.tbi.univie.ac.at/cgi-bin/RNAWebSuite/RNAfold.cgi) that predicts stem-loop and hairpin formation in the CRISPR RNA (crRNA) and transactivating crRNA (tracrRNA) sequences that harbor gRNAs and gRNA scaffolds.

### Construction of CRISPR/Cas9 cassette and transformation into *A. thaliana*

Two gRNA spacer sequences were assembled into the CRISPR/Cas9 construct as described in our earlier study [2]. Briefly, gRNA spacer sequences and *Bsa*I restriction enzyme recognition sites were incorporated into the PCR sense and antisense primers; four primers containing overlapping sequences were used (primer details given in supplementary Table 1). Using the pCBC vector (Addgene) as the template, a single PCR fragment was amplified that contained target 1 gRNA spacer (20 bp), gRNA scaffold (76 bp), *A. thaliana* U6 terminator, promoter, and target 2 gRNA spacer (20 bp). Subsequently, gel-purified PCR fragment was cloned into the multiple cloning site (MCS) of Cas9-expressing binary vector pHEE401 (Addgene) by following the Golden Gate assembly procedure ([54, 55]; supplementary Fig. 8). The recombinant Cas9 editor plasmid (pHEE401:AtAAP6-cr) contained two gRNA expression cassettes driven by the U6 promoter and terminator. pHEE401 harbors the codon-optimized Cas9, whose expression is driven by the *Arabidopsis* egg cell-specific promoter [55]. pHEE401:AtAAP6-cr was transformed into *E. coli* DH5α cells, followed by *R. radiobacter* strain GV3101 as explained above. The length and orientation of the gene constructs were verified via colony PCR (supplementary Fig. 8) and Sanger sequencing. A month-old *A. thaliana* Col-0 wild-type plants growing in pots were transformed with *R. radiobacter* harboring pHEE401:AtAAP6-cr by the floral dip method. T_0_ seeds were germinated in MS medium containing hygromycin at 25 mg L^− 1^. Antibiotic-resistant transformed plants were transplanted into the pots containing soil rite. Plants were grown in the National Phytotron Facility at 21ºC, 60% RH with 16 h light/ 8 h dark at 150 µmol photons m^− 2^ s^− 1^.

### Genotyping of CRISPR-edited plants

In order to detect induced mutations at targeted sites of *AtAAP6*, genomic DNA was isolated from T_0_ plants using the NucleoSpin Plant II Kit (TaKaRa). The targeted genomic region was PCR-amplified using primers flanking the targets 1 and 2 (primer details given in supplementary Table 1) and Sanger sequenced. Sequencing data were analyzed in the SnapGene viewer, mutation types were detected, and mutation efficiency was determined. Homozygous T_0_ lines were selfed to generate T_1_ plants, which were then selfed to obtain T_2_ seeds.

To identify ‘Cas9-free’ plants, genomic DNA was isolated from T_2_ plants using the NucleoSpin Plant II kit (TaKaRa). Next, primers (details given in supplementary Table 1) specific to the *Cas9* gene and the *A. thaliana* housekeeping gene *18 S rRNA* (used as the reference; NCBI Genbank ID: X16077) were used in a multiplex PCR reaction to determine the transgenic elements present in the *A. thaliana* genomic DNA.

### Phenotyping of CRISPR-edited plants

‘Cas9-free’ homozygous T_3_ plants were grown in MS agar in Petri plates. Fortnight-old seedlings were transplanted into pots containing 500 g soil rite, as explained earlier. Plants of 3-weeks-olds were inoculated with 1000 *M. incognita* J2s in the vicinity of the root zone. Plants were periodically watered and provided with Hoagland’s solution as nutrients. At 30 dpi, plants were harvested, and different infection parameters were assessed, as depicted in the earlier section. Roots were stained with acid fuchsin [56] to visualize the endoparasitic nematodes. Additionally, plant morphological characteristics, including plant dry weight, root length, plant height, and average flowering time, were compared between wild-type and edited lines to investigate whether any pleiotropic effects occurred due to targeted mutagenesis.

### Statistical analysis

Data are presented as the mean ± standard errors of at least three independent experiments. Data from different experiments were checked for normality using the Shapiro-Wilk test and then subjected to a one-way or two-way analysis of variance (ANOVA) test in SAS v. 14.1 software. For multiple comparisons across the different treatments, Tukey’s honest significant difference (HSD) test was performed. For pairwise comparison between two treatments, a *t*-test was performed.

### Electronic supplementary material

Below is the link to the electronic supplementary material.

Supplementary Material 1

Supplementary Material 2

## Data Availability

The datasets generated and analyzed in the manuscript are either available in the supplementary files or NCBI Genbank repository (https://www.ncbi.nlm.nih.gov/genbank/) and EnsemblPlants repository (https://plants.ensembl.org/Arabidopsis_thaliana/Info/Index). Any additional data will be available upon request to the corresponding author. Accession numbers of the datasets analyzed in the present manuscript are: AT5G49630, AT1G58360, AT1G10010, AT5G23810, AT1G77380, AT5G63850, AT1G44100, AT5G09220, NP_199774, KAG7612425, XP_020889280, XP_006280391, XP_010442428, XP_009127448, XP_013621160, KAG2312573, KAJ0242994, XP_018443788, NP_001302496, KAF8104771, CAA7021777, CAE6231041, XP_010556498, XP_017610978, KAG7944999, XP_012449709, TYI75790, XP_016690089, XP_021284124, GMJ15248, KAB2030362, XP_039022024, XP_018822119, XP_007018375, TYI82584, XP_023892377, TYH72176, XP_022768570, KAG8491927, MBA0869338, KAJ4701540, XP_030946989, KAF3973726, XP_050264838, XP_059631199, KAH1121762, KAB1212116, XP_019261028, KAA8528415, XP_009793584, XP_016449434, XP_019156541, XP_038716191, XP_059454276, KAH7847261, XP_057972905, GKV33500, KAA3466486, XP_022879119, MBA0607100, and XP_015647443.
