# Supplementary material for: CRISPR/Cas9-induced knockout of an amino acid permease gene (AAP6) reduced Arabidopsis thaliana susceptibility to Meloidogyne incognita
Source: BMC Plant Biol. 2024 Jun 8;24:515. doi: 10.1186/s12870-024-05175-5 (PMC11162074; doi:10.1186/s12870-024-05175-5)

Supplementary Figure 1. Amino acid sequence alignment of *A. thaliana* AAP6 and AAP1 protein using Clustal Omega multiple sequence alignment tool with default settings. * and **:** indicate the identical and similar amino acids, respectively.

```
AAP6      ME----KKKSMFVEQSFPEHEIGDTNKNFDEDGRDKRTGTWMTGSAHIITAVIGSGVLSL   56
AAP1      MKSFNTEGHNHSTAESGDAYTVSDPTKNVDEDGREKRTGTWLTASAHIITAVIGSGVLSL   60
          *:    : :.  . :*   : :.* .**.*****:******:*.****************

AAP6      AWAIAQLGWVAGPAVLMAFSFITYFTSTMLADCYRSPDPVTGKRNYTYMEVVRSYLGGRK   116
AAP1      AWAIAQLGWIAGTSILLIFSFITYFTSTMLADCYRAPDPVTGKRNYTYMDVVRSYLGGRK   120
          *********:** ::*: *****************:*************:**********

AAP6      VQLCGLAQYGNLIGITIGYTITASISMVAVKRSNCFHKNGHNVKCATSNTPFMIIFAIIQ   176
AAP1      VQLCGVAQYGNLIGVTVGYTITASISLVAVGKSNCFHDKGHTADCTISNYPYMAVFGIIQ   180
          *****:********:*:*********:*** :*****.:**...*: ** *:* :*.***

AAP6      IILSQIPNFHNLSWLSILAAVMSFCYASIGVGLSIAKAAGGGEHVRTTLTGVTVGIDVSG   236
AAP1      VILSQIPNFHKLSFLSIMAAVMSFTYATIGIGLAIATVAGG-KVGKTSMTGTAVGVDVTA   239
          :*********:**:***:****** **:**:**:**..*** :  :*::**.:**:**:.

AAP6      AEKIWRTFQAIGDIAFAYAYSTVLIEIQDTLKAGPPSENKAMKRASLVGVSTTTFFYMLC   296
AAP1      AQKIWRSFQAVGDIAFAYAYATVLIEIQDTLRSS-PAENKAMKRASLVGVSTTTFFYILC   298
          *:****:***:*********:**********::. *:********************:**

AAP6      GCVGYAAFGNDAPGNFLTGFGFYEPFWLIDFANVCIAVHLIGAYQVFCQPIFQFVESQSA   356
AAP1      GCIGYAAFGNNAPGDFLTDFGFFEPFWLIDFANACIAVHLIGAYQVFAQPIFQFVEKKCN   358
          **:*******:***:***.***:**********.*************.********.:.

AAP6      KRWPDNKFITGEYKIHVPCCGDFSINFLRLVWRTSYVVVTAVVAMIFPFFNDFLGLIGAA   416
AAP1      RNYPDNKFITSEYSVNVPFLGKFNISLFRLVWRTAYVVITTVVAMIFPFFNAILGLIGAA   418
          :.:*******.**.::**  *.*.*.::******:***:*:********** :*******

AAP6      SFWPLTVYFPIEMHIAQKKIPKFSFTWTWLKILSWTCFIVSLVAAAGSVQGLIQSLKDFK   476
AAP1      SFWPLTVYFPVEMHIAQTKIKKYSARWIALKTMCYVCLIVSLLAAAGSIAGLISSVKTYK   478
          **********:******.** *:*  *  ** :.:.*:****:*****: ***.*:* :*

AAP6      PFQAP--      481
AAP1      PFRTMHE      485
          **::
```

Supplementary Figure 2. The secondary structure prediction of designed sgRNAs. Both the sgRNAs contained the maximum free guide sequence (minimal internal base pairing in the guide sequence leads to greater target recognition), an intact tetra loop (repeat crRNA and anti-repeat tracrRNA), stem loop 2 and 3 (in tracrRNA). The intact stem loop structures promote stable Cas9-sgRNA-DNA complex formation and improve *in vivo* editing efficacy.

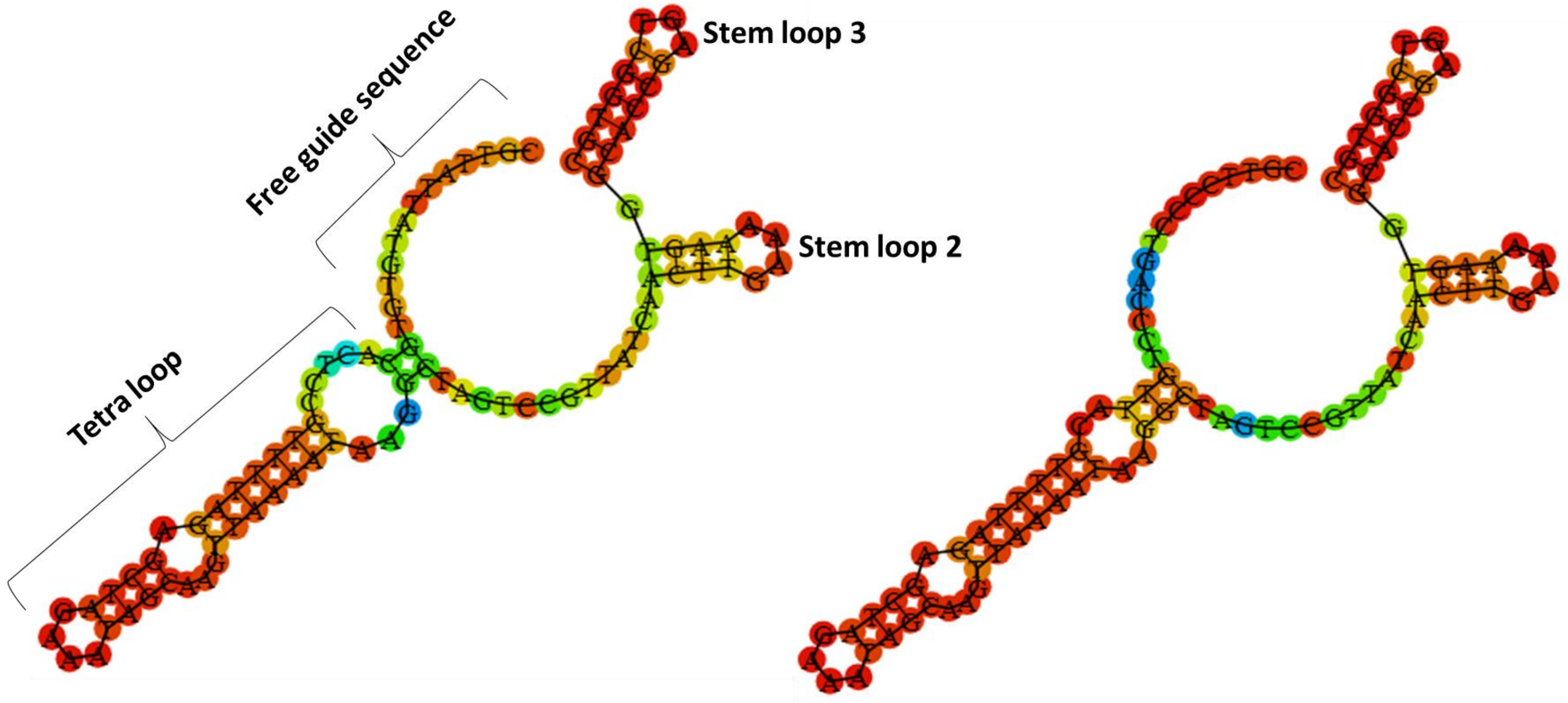

gRNA1: CGTTATTATGTGTGCACTCC + Scaffold

gRNA2: CGTTCCCCTGACCCTGTTAC + Scaffold

Scaffold: GTTTTAGAGCTAGAAATAGCAAGTTAAAATAAGGCTAGTCCGTTATCAACTTGAAAAAGTGGCACCGAGTCGGTGC

Supplementary Figure 3. Sequence chromatograms showing CRISPR/Cas9-induced mutations (indicated by red arrows) in the two target sites of *AtAAP6* genomic sequence in different genome-edited lines. PAM sequences are underlined in red.

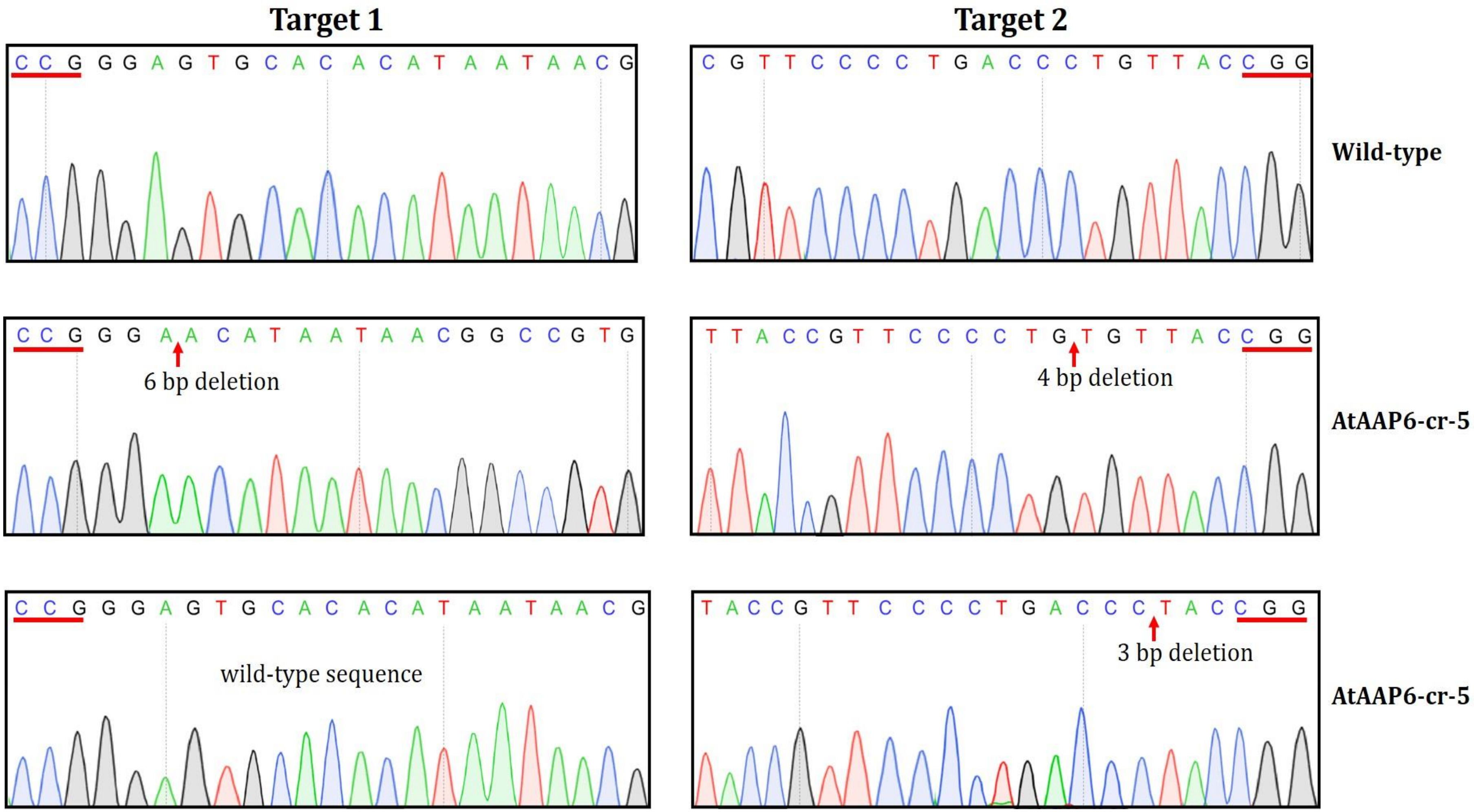

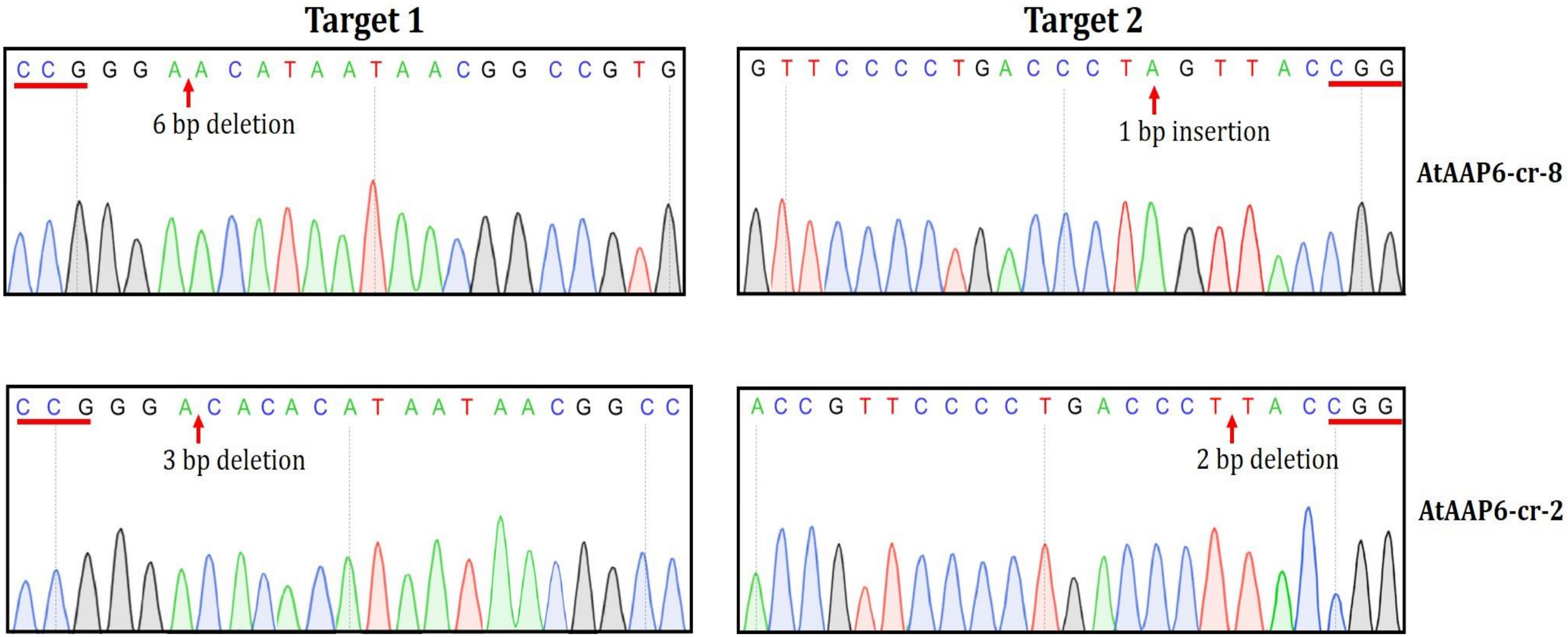
Target 1
Target 2
C C G G G A A C A T A A T A A C G G C C G T G
6 bp deletion
G T T C C C C T G A C C C T A G T T A C C G G
1 bp insertion
AtAAP6-cr-8
C C G G G A C A C A C A T A A T A A C G G C C
3 bp deletion
A C C G T T C C C C T G A C C C T T A C C G G
2 bp deletion
AtAAP6-cr-2

Supplementary Figure 4. RT-qPCR-based expression analysis of *AAP6* gene and its homologues (*AAP1*, *AAP2*, *AAP3*, *AAP4*, *AAP5*, *AAP7*, and *AAP8*) in genome edited line AtAAP6-cr-5 and wild-type plants. Fold change in expression of target gene was set as 1 in wild-type, and statistically compared with the expression in edited plants. Asterisk indicates significant difference (Tukey's HSD test, $P < 0.01$) in edited plant compared to the wild-type. Gene expression was normalized using two housekeeping genes of *A. thaliana* (ubiquitin and *18S rRNA*). Each bar represents the mean fold change value ± standard error (SE) of qPCR runs in five biological and three technical replicates.

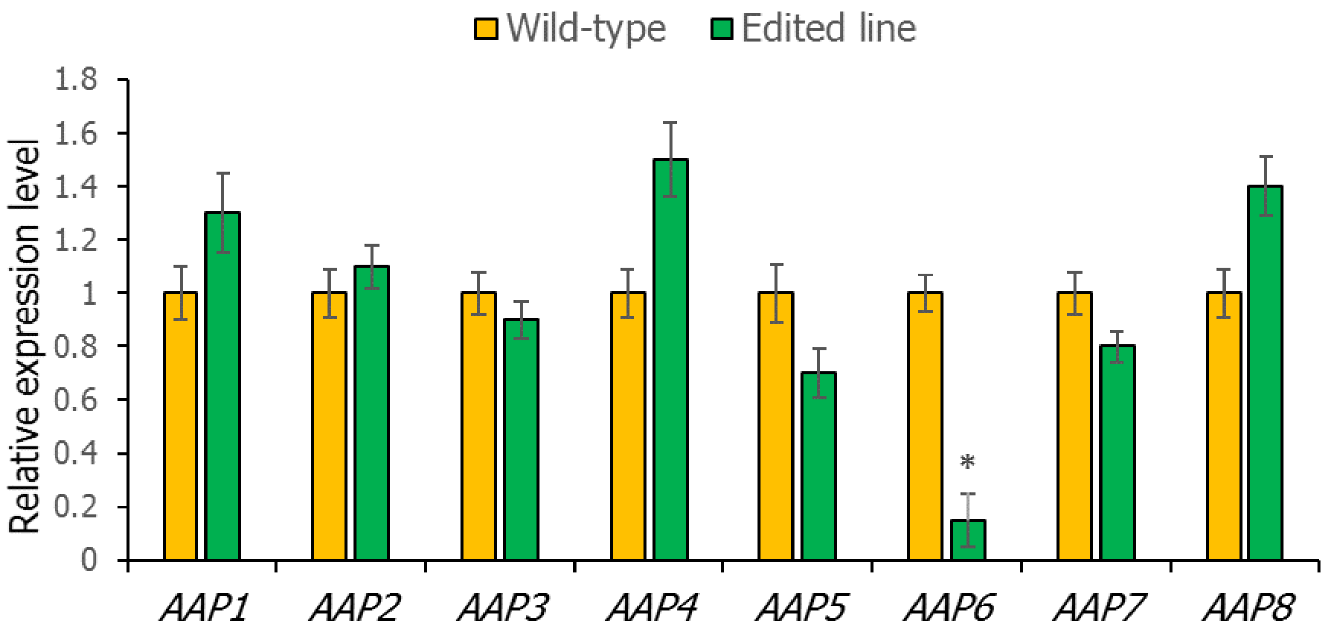

Supplementary Figure 5. Detection of 'Cas9-free' homozygous *AtAAP6* edited plants in the T_2 generation. The partial coding sequence of *Cas9* gene (789 bp) was detected in different mutant individuals. *Arabidopsis thaliana 18S rRNA* gene (506 bp) was used as the reference in multiplex PCR reactions. M – 100 bp DNA ladder, WT – wild-type plants, V – plasmid vector, N – negative control sans template DNA.

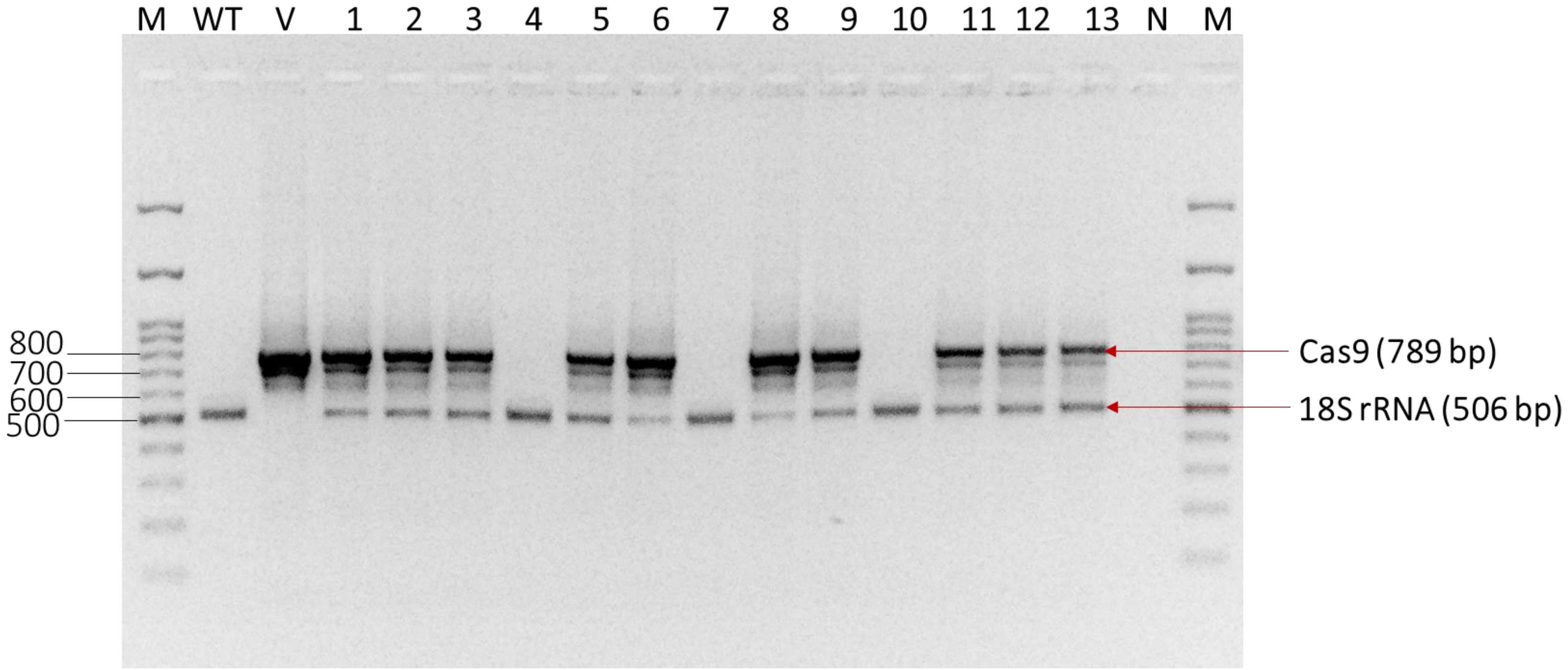

Supplementary Figure 6. Comparative phenotyping of *A. thaliana* wild-type and AAP6 mutant line for different growth parameters including average dry weight (g) and root length (cm) of 14-day-old seedling (grown in MS media), average flowering time (days), and average height (cm) of 30-day-old plant (grown in soil). Bars represent mean ± SE (n = 20). No significant difference was observed between wild-type and mutant line across the different parameters; Tukey's HSD test, $P > 0.01$.

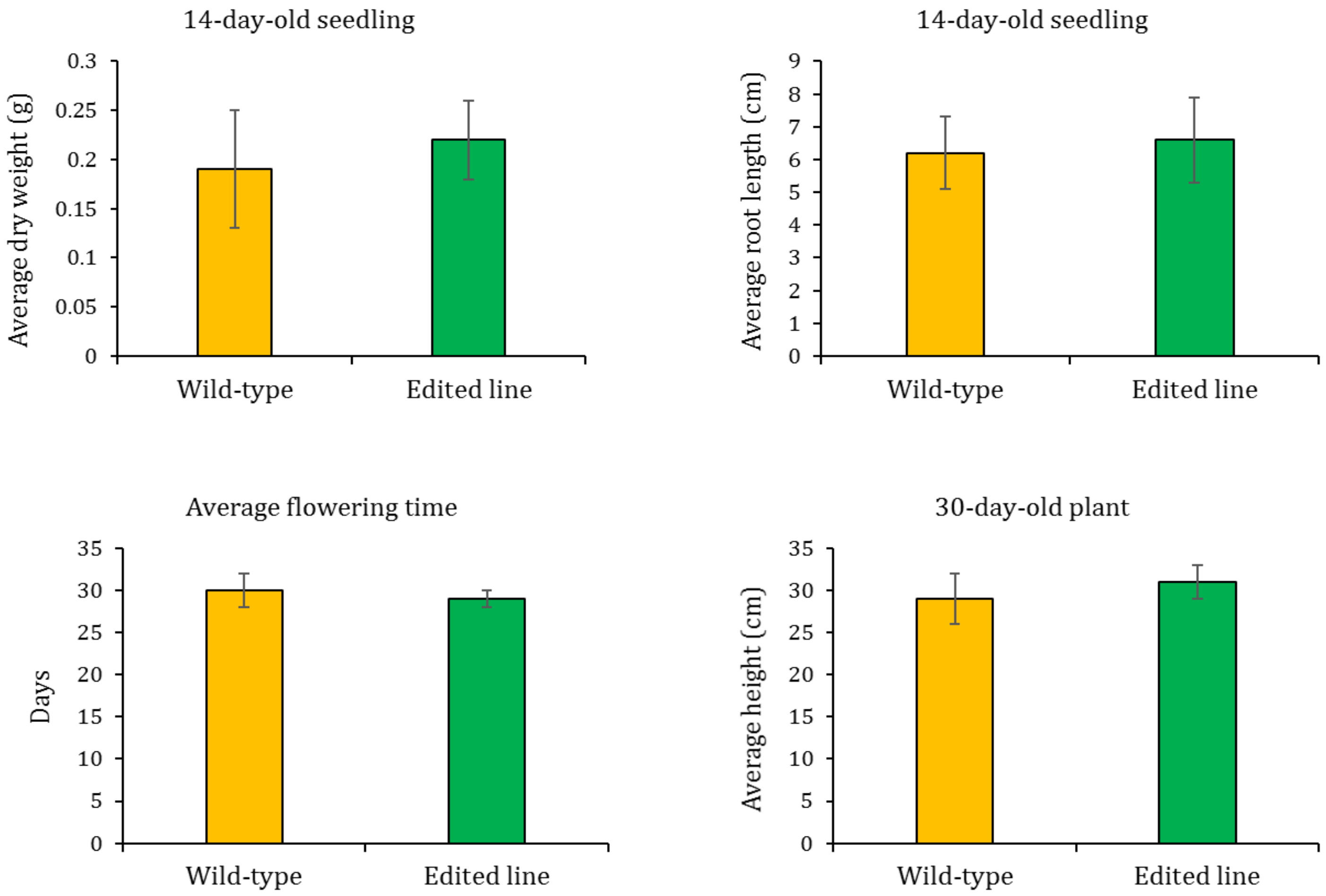

Supplementary Figure 7. RT-qPCR-based expression analysis of defense response genes in roots **(A)** and shoots **(B)** of *M. incognita*-infected *A. thaliana* wild-type and AAP6 mutants at 3 days after inoculation. Fold change in expression was set as 1 in wild-types and statistically compared with expression in AAP6 mutants (no significant difference was observed; Tukey's HSD test, $P > 0.01$). Gene expression was normalized using two housekeeping genes of *A. thaliana* (ubiquitin and *18S rRNA*). Each bar represents the mean fold change value ± standard error (SE) of qPCR runs in five biological and three technical replicates.

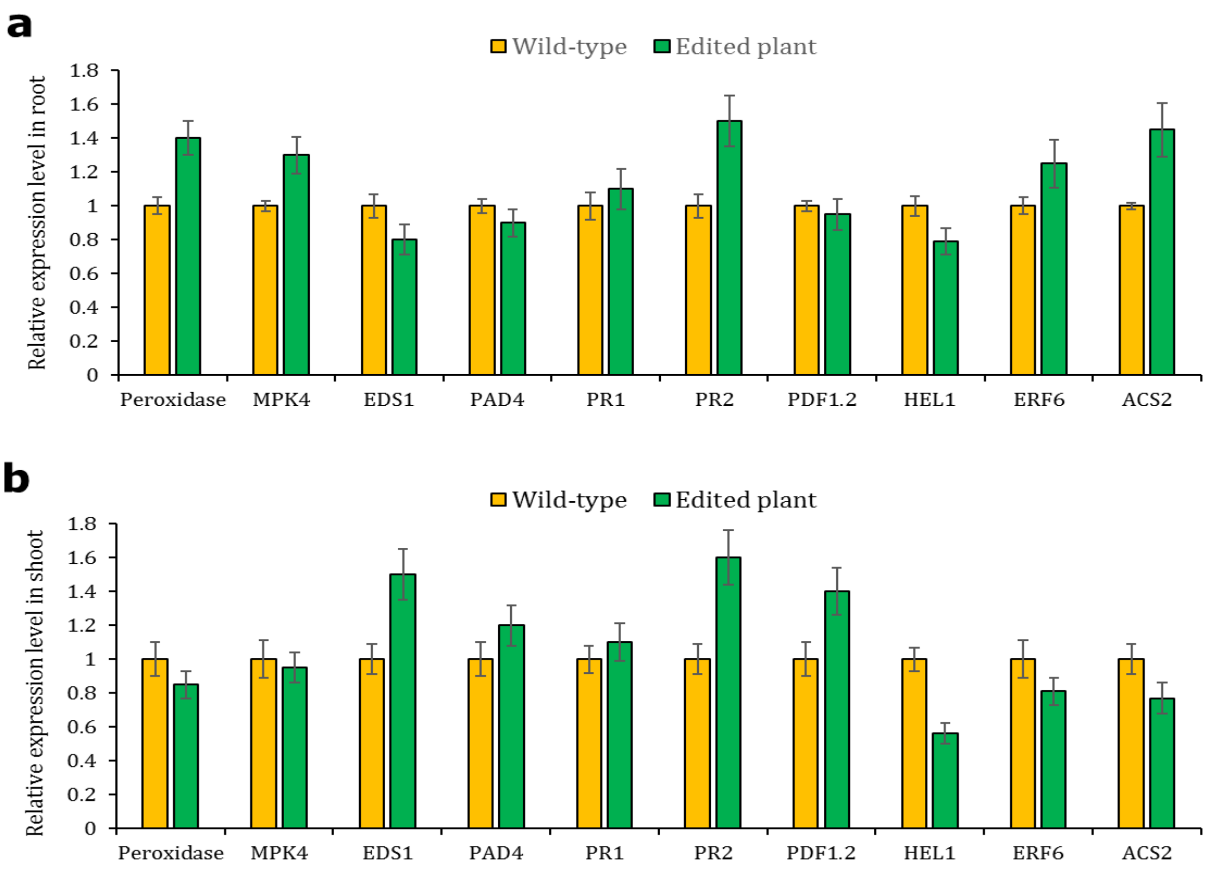

Supplementary Figure 8. CRISPR/Cas9 editing assembly is schematically represented. Plasmid pCBC was used as the template to generate a PCR product containing the two targets or gRNA spacers of *AAP6* gene (flanked by the *Bsa*I endonuclease sites), gRNA scaffold, *Arabidopsis* U6 gene terminator (U6-26t) and promoter (U6-29p). PCR forward/sense (F) and reverse/antisense (R) primers contained the two target sequences and *Bsa*I site. Two gRNA expression cassettes were assembled into the T-DNA region of Cas9-expressing binary vector pHEE401 via Golden Gate cloning by replacing the spectinomycin resistance (SpecR) gene. In recombinant pHEE401 (pHEE401:AtAAP6-cr), first gRNA expression cassette is driven by the *Arabidopsis* U6 promoter U6-26p. Codon optimized Cas9 expression is driven by an egg cell-specific promoter (EC1p) and *rbcS E9* terminator (rbcS-E9t). NLS, nuclear localization signal; 35Sp, CaMV35S promoter; HygR, Hygromycin resistance; PolyA, CaMV35S terminator; LB, left border; RB, right border. Bottom panel depicts the nucleotide sequence of two gRNA expression cassettes generated in the present study. Gel photograph indicates PCR amplification of expected 826 bp fragment from different colonies of *Rhizobium radiobacter* GV3101 harboring the pHEE401:AtAAP6-cr construct.

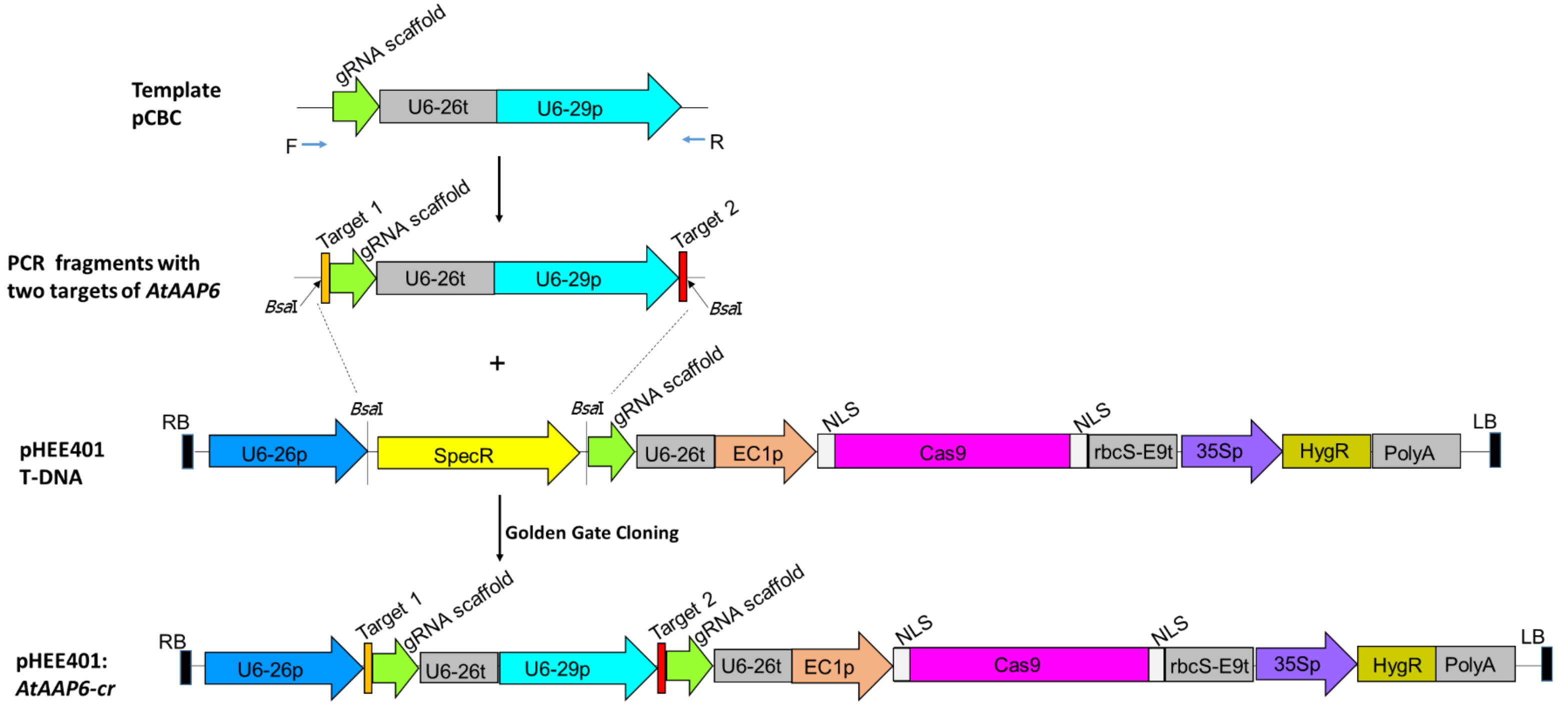

(U6-26p)-(Target-1)-(sgRNA-scaffold)-(U6-26t)-(U6-29p)-(Target-2)-(sgRNA-scaffold)-(U6-26t)

CGACTTGCCTTCCGCACAATACATCATTTCTTCTTAGCTTTTTTTCTTCTTCTTCGTTCATACAGTTTTTTTTTGTTTATCAGCTTACATTTTCTTGAA
CCGTAGCTTTCGTTTTCTTCTTTTTAACTTTCCATTCGGAGTTTTTGTATCTTGTTTCATAGTTTGTCCCAGGATTAGAATGATTAGGCATCGAACC
TTCAAGAATTTGATTGAATAAAACATCTTCATTCTTAAGATATGAAGATAATCTTCAAAAGGCCCCTGGGA**ATCTGAAAGAAGAGAAGCAGGC**
CCATTTATATGGGAAAGAACAATAGTATTTCTTATATAGGCCCATTTAAGTTGAAAACAATCTTCAAAAGTCCCACATCGCTTAGATAAGAAAAC
GAAGCTGAGTTTATATACAGCTAGAGTCGAAGTAGTGATTGCGTTATTATGTGTGCACTCCGTTTTAGAGCTAGAAATAGCAAGTTAAAATAAGG
CTAGTCCGTTATCAACTTGAAAAAGTGGCACCGAGTCGGTGCTTTTTTTTGCAAAATTTTCCAGATCGATTTCTTCTTCCTCTGTTCTTCGGCGTTC
AATTTCTGGGGTTTTCTCTTCGTTTTCTGTAACTGAAACCTAAAATTTGACCTAAAAAAAATCTCAAATAATATGATTCAGTGGTTTTGTACTTTTC
AGTTAGTTGAGTTTTGCAGTTCCGATGAGATAAACCAATATTAATCCAAACTACTGCAGCCTGACAGACAAATGAGGATGCAAACAATTTTAAA
GTTTATCTAACGCTAGCTGTTTTGTTTCTTCTCTCTGGTGCACCAACGACGGCGTTTTCTCAATCATAAAGAGGCTTGTTTTACTTAAGGCCATAAT
GTTGATGGATCGAAAGAAGAGGGCTTTTAATAAACGAGCCCGTTTAAGCTGTAAACGATGTCAAAAACATCCCACATCGTTCAGTTGAAAATAG
AAGCTCTGTTTATATATTGGTAGAGTCGCTAAGAGATTGCGTTCCCCTGACCCTGTTACGTTTTAGAGCTAGAAATAGCAAGTTAAAATAAGGCT
AGTCCGTTATCAACTTGAAAAA**GTGGCACCGAGTCGGTGC**TTTTTTTTGCAAAATTTTCCAGATCGATTTCTTCTTCCTCTGTTCTTCGGCGTTC
AATTTCTGGGGTTTTCTCTTCGTTTTCTGTAACTGAAACCTAAAATTTGACCTAAAAAAAATCTCAAATAATATGATTCAGTGGTTTTGTACTTTTC
AGTTAGTTGAGTTTTGCAGTTCCGATGAGATAAACCAATA

Bold letters represent primer-binding sequences for colony PCR.

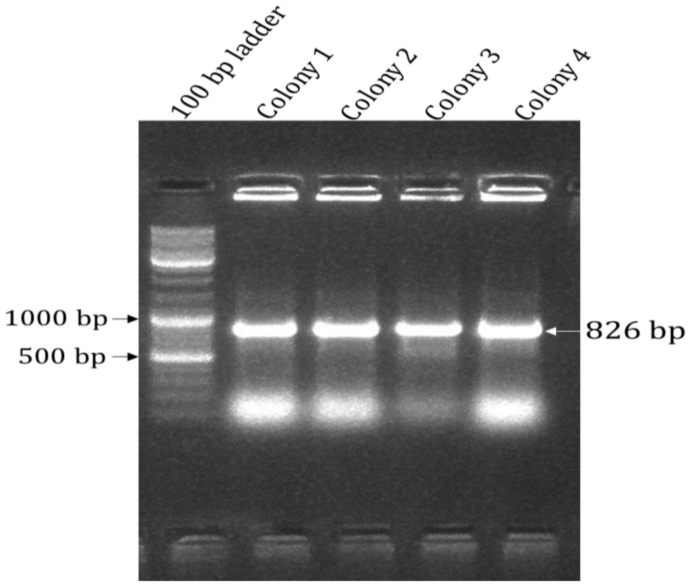

Supplement: Supplementary file 1 — Supplementary Material 1 [file 12870_2024_5175_MOESM1_ESM.pdf]
