# Supplementary material for: CRISPR/Cas9-induced knockout of an amino acid permease gene (AAP6) reduced Arabidopsis thaliana susceptibility to Meloidogyne incognita
Source: BMC Plant Biol. 2024 Jun 8;24:515. doi: 10.1186/s12870-024-05175-5 (PMC11162074; doi:10.1186/s12870-024-05175-5)
Supplement: Supplementary file 2 — Supplementary Material 2 [file 12870_2024_5175_MOESM2_ESM.pdf]

Supplementary Table 1. List of primers used for CRISPR/Cas9 experiment. Annealing temperature – 60ºC. Underlined sequences are *Bsa*I recognition site. Smaller case letters are gRNA spacer or target sequences.

**Primer name**	**Sequence (5´- 3´)**	**Purpose**
pCXSN:AAP6_F	ATGGAGAAGAAGAAGAGCATGT	Overexpression of *AtAAP6* gene
pCXSN:AAP6_R	CTAAGGAGCCTGGAAAGGCT	
pCXGUS-P:AAP6_F	TTGTAGTGACTGGCTAAACCG	Overexpression of *AtAAP6* promoter:GUS fusion
pCXGUS-P:AAP6_R	GAAGAGGCCAAAGGTAGTATG	
qPCR-AAP6_F	AACCATGCTTGCCGATTGTT	qPCR analysis of *AtAAP6* gene
qPCR-AAP6_R	TCTTCACTGCCACCATGCTA	
qPCR-UBQ_F	GGAAAGACCATCACCCTTGA	*A. thaliana* ubiquitin gene as internal control in qPCR
qPCR-UBQ_R	ATCCTCAAGCTGCTTTCCAG	
qPCR-18S_F	GGTGGTAACGGGTGACGGAGAAT	*A. thaliana 18S rRNA* gene as internal control in qPCR
qPCR-18S_R	CGCCGACCGAAGGGACAAGCCGA	
Target1_Bsa_F	ATATATGGTCTCGATTGcgttattatgtgtgcactccGTT	gRNA1 and gRNA2 cassette construction for CRISPR/Cas9 experiment
Target1_gRNA scaffold_F	TGcgttattatgtgtgcactccGTTTTAGAGCTAGAAATAGC	
Target2_U6-29p_R	AACgtaacagggtcaggggaacgCAATCTCTTAGTCGACTCTAC	
Target2_Bsa_R	ATTATTGGTCTCGAAACgtaacagggtcaggggaacgCAA	
Upstream Target 1_F	TTCTACGTGTTAATGCGGACA	PCR amplification of *AtAAP6* target 1 and 2 (mutation detection)
Downstream Target 2_R	GGTTCTTCAGAGTGATTGACCA	
Cas9_F	CCTGATCTACCTCGCTCTGG	PCR-based detection of Cas9 protein encoding gene in mutants
Cas9_R	TGAATCTGGTGTGGGATGCT	
At_18S_F	ATTAACAGGGACAGTCGGGG	PCR-based detection of *18S rRNA* gene in mutants
At_18S_R	GATGCCTCCACGTAGCTAGT	
Peroxidase_F	ACGTGGAATTGGGAAGGCTA	qPCR analysis of defense response genes
Peroxidase_R	TGCGCGAATCCTAATGTGTG	
MPK4_F	TAAGCCCAGCGTAACAGTGA	

MPK4_R	ACGTCTTAGAGATCAGCGGG	
EDS1_F	TACCTTGAGCCTCGTTGTGT	
EDS1_R	GGGCAAGAACATGAGGCAAA	
PAD4_F	TCCTCTGCTCGGAAACCAAT	
PAD4_R	GAGTTGCTGTGGTGTTGAGG	
PR1_F	TGCTCTTGTTCTTCCCTCGA	
PR1_R	CTAACCCACATGTTCACGGC	
PR2_F	TCCGGTACATCAACGTTGGA	
PR2_R	AAGGGAGATTGCTTGCTTGC	
PDF1.2_F	TCTCTTTGCTGCTTTCGACG	
PDF1.2_R	ACTTGTGTGCTGGGAAGACA	
HEL1_F	CACGTGGGATGCTGATAAGC	
HEL1_R	CATCCAAATCCAAGCCTCCG	
ERF6_F	CCTACTACTGCCACCACCAA	
ERF6_R	ACAGTAACGCGAGGAGGATT	
ACS2_F	ATGTGTCTCCTGGCTCTTCC	
ACS2_R	GTCTGCGTCCATTTCAGCTT	
qPCR-AAP1_F	CTTCACCTCCACCATGCTTG	qPCR analysis of *AtAAP1* gene
qPCR-AAP1_R	GCCCTTTATCGTGGAAGCAG	
qPCR-AAP2_F	AGTCTTTGCTCAGCCCATCT	qPCR analysis of *AtAAP2* gene
qPCR-AAP2_R	ACGACAAAGCCACTCCTGTA	
qPCR-AAP3_F	AACCACCAAACAGTTCTCGC	qPCR analysis of *AtAAP3* gene
qPCR-AAP3_R	CGGCAGAGAAGAGCAACATC	
qPCR-AAP4_F	ATGGCGATCAAGAGGTCCAA	qPCR analysis of *AtAAP4* gene
qPCR-AAP4_R	AGGACATGATAGCAGCGACA	

qPCR-AAP5_F	ACTGGAGTCACTGTTGGGAC	qPCR analysis of *AtAAP5* gene
qPCR-AAP5_R	ACTGTAGTCACTGCCACACT	
qPCR-AAP7_F	AACACCAACCGAGCTTCAAC	qPCR analysis of *AtAAP7* gene
qPCR-AAP7_R	CAGGGAAACGGTAGCAATCG	
qPCR-AAP8_F	GCCCACCAGAGAACAAAGTG	qPCR analysis of *AtAAP8* gene
qPCR-AAP8_R	GATGGAGAGCAATGCAAGCA	
